# Common mycorrhizal networks enhance defense responses against pathogens in neighboring plants

**DOI:** 10.1002/imo2.46

**Published:** 2024-12-07

**Authors:** Yingde Li, Yong Wei, Youlei Shen, Rongchun Zheng, Yajie Wang, Tingyu Duan, Zhibiao Nan

**Affiliations:** ^1^ State Key Laboratory of Herbage Improvement and Grassland Agro‐ecosystems Lanzhou University Lanzhou China; ^2^ Key Laboratory of Grassland Livestock Industry Innovation Ministry of Agriculture and Rural Affairs Lanzhou China; ^3^ Engineering Research Center of Grassland Industry Ministry of Education, Gansu Tech Innovation Centre of Western China Grassland Industry Lanzhou China; ^4^ College of Pastoral Agriculture Science and Technology Lanzhou University Lanzhou China

**Keywords:** common mycorrhizal networks, defense signals, high−throughput sequencing, phyllosphere microbiome, ryegrass, white clover

## Abstract

Arbuscular mycorrhizal (AM) fungi can establish common mycorrhizal networks (CMNs) and assemble their own phyllosphere microbiome. Although the roles of the AM fungi in soil microbiomes have been extensively studied, little is known about the role of CMNs in collective community−level changes of the phyllosphere microbiome in response to infection by pathogens. Here, we explored the impact of CMNs that linked the donor white clover (*Trifolium repens* L.) plants infected with the pathogen *Stemphylium sarciniforme* on changes in defense compounds and the phyllosphere microbiome in receiver perennial ryegrass (*Lolium perenne* L.). This included examining plant defense enzymes, phytohormones, phyllosphere microbiome diversity, community structure, and network complexity. The donor clover infected with the pathogen and its linked receiver ryegrass through the CMNs had a higher activity of catalase along with higher levels of abundance‐based coverage estimato (ACE), genera, and species richness in the receiver ryegrass. The pathogen infection of the donor clover altered the community structure of the phyllosphere microbiome in the receiver ryegrass, and the fungal network was significantly more complex. The activities of catalase and polyphenol oxidase exhibited a positive correlation with the alpha−diversity of the ryegrass phyllosphere fungi and also showed a significant positive relationship with the relative abundance of microbes associated with resistance to disease. In summary, this study revealed differences in the phyllosphere microbiomes of receiver ryegrass linked through CMNs to pathogen−infected and uninfected donor clovers. These changes in the phyllosphere microbiomes correlated with elevated levels of defense enzymes in the receiver ryegrass linked by white clover infected with pathogens, thus, potentially playing a role in plant defense against pathogens. These findings provide novel insights into the role of CMNs in reshaping phyllosphere microbiomes at the plant community levels under pathogen invasion.

## Highlights

- Common mycorrhizal networks (CMNs) provide a channel for interplant communication, which enables plants of different species to prepare for pathogen infections without direct exposure.
- CMNs change the microbial abundance and community structure of the neighboring plants following pathogen infection in the donor plants, enhancing microbial interaction and network complexity in neighboring plants.
- This activation may trigger defense responses in the receiver plants against potential pathogens, which enhances the system's ability to resist infections.

## Graphical abstract

The common mycorrhizal networks (CMNs) provide a channel for interplant communication, which enables plants of different species to prepare for pathogen infections without direct exposure. The CMNs change the microbial abundance and community structure of the neighboring plants following pathogen infection in the donor plants, which further enhances microbial interaction and network complexity in neighboring plants. This activation may trigger defense responses in the receiver plants against potential pathogens, which enhances the system's ability to resist infections.

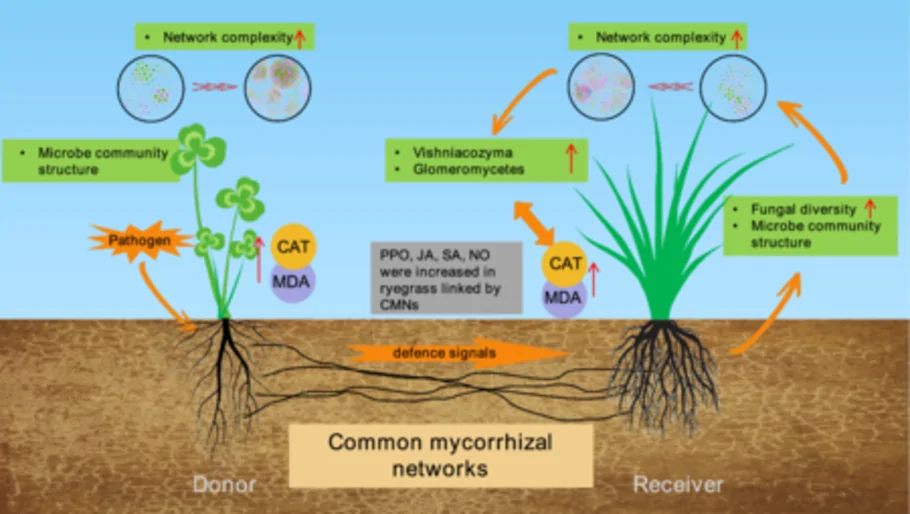

## 1 INTRODUCTION

Arbuscular mycorrhizal (AM) fungi are involved in symbiotic relationships with various herbaceous plants and are widely distributed globally. They are one of the most functionally important groups of soil microbes [1]. AM fungi play a significant role in improving the uptake of mineral nutrients by host plants [2, 3] and can enhance the tolerance of the host to pathogens [4] and insect pests [5]. AM fungi lack specificity to their host plants [1], thereby external mycelia known as ‘common mycelial networks’ (CMNs) connect the roots of various plant species, as well as individuals of the same species [6, 7]. CMNs can regulate the competition among plant individuals and redistribute their nutrient resources. For example, CMNs extend the bioactive zone of allelochemicals released by marigolds (*Tagetes tenuifolia* Cav.) in the soil, which inhibits the growth of neighboring plants [8]. CMNs facilitate the transport of mineral nutrients, such as soil nitrogen and phosphorus (P), between plants [7]. In addition to transferring mineral nutrients between plants, the CMNs can also transfer stress defense signals [9, 10]. Previous findings indicate that the CMNs transfer defense signals between plants when they encounter stressors, such as pathogen infection and insect feeding. This transfer induces defense responses from the neighboring plants and can effectively serve as an early “warning” before the stressor fully manifests [11, 12, 13]. CMNs can swiftly transfer signaling compounds following attacks by plant pathogens [11, 14] or insect pests [15, 16]. Broad beans (*Vicia faba* L.) exposed to pea aphids (*Acyrthosiphon pisum* Harris.) and broad beans connected to the exposed beans by CMNs (aphid−free plants) release similar volatile organic compounds to repel the pea aphids and attract their natural enemies [12]. The CMNs transfer signals from infected to healthy neighboring seedlings that can induce higher levels of salicylic acid (SA) in neighboring plants. This activation of defense responses helps protect these neighboring plants from citrus canker caused by *Xanthomonas axonopodis* [14]. Interplant connections through the CMNs enhance disease resistance and induce the activities of defensive enzymes, including lipoxygenase, phenylalanine ammonia−lyase, peroxidase (POD), and polyphenol oxidase (PPO) in healthy tomato plants (*Lycopersicon esculentum* Mill.) and those connected to plants infected with early blight (*Alternaria solani*) [11]. These findings indicate that the signals for resistance against fungal diseases between plants are transmitted via these networks. Therefore, the CMNs offer significant potential for crop management through this belowground plant−to–plant signaling mechanism [12, 14].

The previous studies focused on the transfer of defense signals mediated by CMNs between plants of the same species, such as *Salvia miltiorrhiza* [17], potato (*Solanum tuberosum* L.) [18], tomato (*Solanum lycopersicum* L.) [19], *Nicotiana attenuata* [20], and trifoliate orange (*Poncirus trifoliata* [L.] Raf) [14]. However, mostly different species of plants coexist in natural ecosystems, and CMNs connect these diverse plant species [6, 21]. For example, flax (*Linum usitatissimum* L.) and sorghum (*Sorghum bicolor* L.) [22], *Leymus chinensis* and *Cleistogene squarrosa* [23], and perennial ryegrass (*Lolium perenne* L.) and white clover (*Trifolium repens* L.) [21] can be interlinked by the CMNs of AM fungi. The CMNs help to transfer nutrients and enhance productivity. In addition, the CMNs may also contribute to facilitating interplant communication and the defense of plants to biotic and abiotic stresses between different species of plants. These findings highlight the importance of CMNs and the urgent need for additional research on their function and role in transferring resistance signals, particularly in exploring strategies for the stability of ecosystems and sustainable agricultural pest and disease management.

The phyllosphere is the largest biological surface on earth and harbors a diverse array of functional microbiomes that play crucial roles in plant growth, stress resistance, and productivity [24]. These microbiomes provide a defense against plant pathogens [25, 26]. For example, the differences in phyllosphere microbiomes between citrus (*Citrus* spp.) leaves infected by the citrus melanose pathogen (*Diaporthe citri*) and uninfected citrus leaves highlighted several bacteria that have been associated with the shift in microbiome composition that positively influenced the performance of plants infested with *D. citri* [27]. *Bacillus* and *Trichoderma* changed the composition and abundance of the phyllosphere bacterial community of common vetch (*Vicia sativa*), and these changes strongly correlated with the activities of plant defense enzymes (PPO) and signaling molecules, such as jasmonic acid (JA) and nitric oxide (NO) [28]. It is well known that AM fungi modulate the microbes in the plant rhizosphere [29, 30]. The plant roots and rhizosphere share microbial populations with the aboveground parts of the plant and serve as crucial contributors to the phyllosphere microbiome [31]. Notably, water stress and the disruption of mycorrhizal associations changed the composition of phyllosphere microbiome [32]. AM fungi significantly increased the diversity of endophytic microbial communities of maize (*Zea mays* L.) [33]. However, the effect of AM fungi on the plant phyllosphere microbiota remains poorly understood. Importantly, existing studies have not yet explored the changes in the phyllosphere microbiome modulated by CMNs, particularly during biotic stresses, such as pathogen invasion.

Perennial ryegrass (*L. perenne*) and white clover (*T. repens*) are both crucial high−quality forage and turf species. The mixed cultivation of the two species is common in the management and establishment of pasture [34]. This combination represents a blend of legumes and grasses that are frequently utilized in revegetation practices. Notably, previous studies demonstrated that the establishment of CMNs between white clover and ryegrass resulted in significant changes in plant growth, arsenic uptake, phosphorus nutrition, and competitive interactions [21]. However, studies have not explored the transmission of disease resistance signals between white clover and ryegrass through CMNs. Here, we aimed to explore the effects of pathogen infection of the donor white clover on the defense compounds and phyllosphere microbiome of the receiver ryegrass linked to the CMNs. This includes examining defense enzymes, microbial diversity, community structure, and microbial network complexity, among other factors, while revealing the correlation between defense compounds and the phyllosphere microbiome. In addition, if the CMNs act as conduits for signaling compounds between white clover infected with a pathogen and uninfected ryegrass, we hypothesized the following: (1) ryegrass linked to white clover infected with pathogens by CMNs exhibit defense responses similar to those of the infected white clover, thus, inducing a change in the levels of defense compounds and the phyllosphere microbiome; and (2) CMNs change the microbial diversity and community structure of the phyllosphere, which is related to changes in their defense compounds, and potentially play a role in the plant's defense against pathogens.

## 2 RESULTS

### Mycorrhizal colonization and plant growth

The mycorrhizae colonized both the AM−L− (the receiver ryegrass was inoculated with the AM fungus and was not linked with donor clover through CMNs) and NM−L+ (the receiver ryegrass was not inoculated with AM fungus and was linked with donor clover through CMNs) perennial ryegrass treatment groups. We observed that the AM fungal hyphae formed CMNs belowground. They passed through the 37 μm nylon mesh and connected the donor and receiver plants. The percentage of mycorrhizal colonization in perennial ryegrass ranged from 70% to 95% in both the AM−L− treatments and NM−L+ treatments (Figure S1). No significant differences were observed in plant biomass indicators across the treatments, except for a reduction in donor clover root biomass in the pathogen−infected group (Figure S1).

### Plant defense enzymes

**Figure 1:**
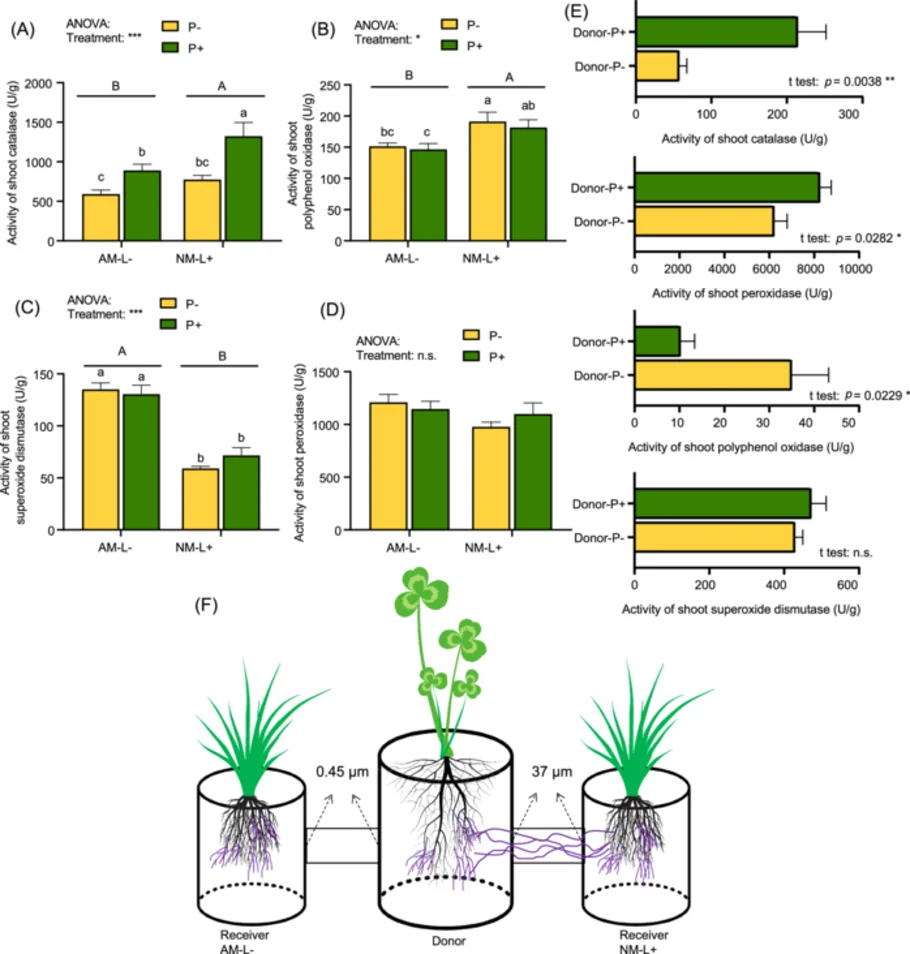
Experimental mesocosm, and defense enzymes of the donor white clover (*Trifolium repens* L.) and receiver perennial ryegrass (*Lolium perenne* L.). (A) Catalase, (B) polyphenol oxidase, (C) superoxide dismutase, and (D) peroxidase activity of perennial ryegrass (*Lolium perenne* L.) in the linking donor white clover (*Trifolium repens* L.) by the common mycorrhizal networks (NM−L +) or the not linked (AM−L−) treatment. (E) Catalase, peroxidase, polyphenol oxidase, and superoxide dismutase activities of white clover; (F) experimental mesocosm (*n* = 5) showing the donor white clover and two receiver perennial ryegrass. All the plants were grown in the mycorrhizal condition, but one plant was prevented from forming mycelial connections to the donor plants (0.45 μm mesh, left). Another was allowed to initially form connections to the donor (37 μm mesh, right). P+ , donor clover infected with the pathogen *Stemphylium sarciniforme*. P‐, donor clover not infected with the pathogen *S. sarciniforme*. Values are presented as the mean ± standard error of mean (SEM) of five replicates. Different lowercase letters above the bars indicate significant differences across the groups and treatments at *p* < 0.05 by least significant difference (LSD) test. Different uppercase letters above the horizontal line indicate significant differences between the AM‐l‐ and NM‐L+ ryegrass at *p* < 0.05. The donor white clover data were analyzed using Student's *t*‐test. *p < 0.05. **p < 0.01. ***p < 0.001. n.s., no significant differences.

In the receiver ryegrass, the presence of CMN connections significantly altered the activities of superoxide dismutase (SOD) (*p* < 0.001), PPO (*p* = 0.002), and catalase (CAT) (*p *= 0.014) (Figure 1A–D). There were significantly higher activities of CAT and POD in the donor clover plants infected with the pathogen (*p* = 0.004 and *p* = 0.028, respectively), whereas there was significantly less activity of PPO (*p *= 0.023) compared to the uninfected clover (Figure 1E). The activities of CAT and PPO were significantly higher in the NM−L+ ryegrass compared to the AM−L− ryegrass (Figure 1B,C). Notably, the ryegrass connected through CMNs had higher levels of CAT activity when the donor clover was infected with the pathogen compared to treatment with healthy clover, which suggested that defense signals may be transferred through the CMNs between white clover and ryegrass (Figure 1A). There were lower levels of SOD in the NM−L+ ryegrass than in the AM−L− ryegrass (Figure 1C). These findings indicate that the CMN connection specifically influenced the enzyme activity of ryegrass.

### Plant hormones and photosynthesis

**Figure 2:**
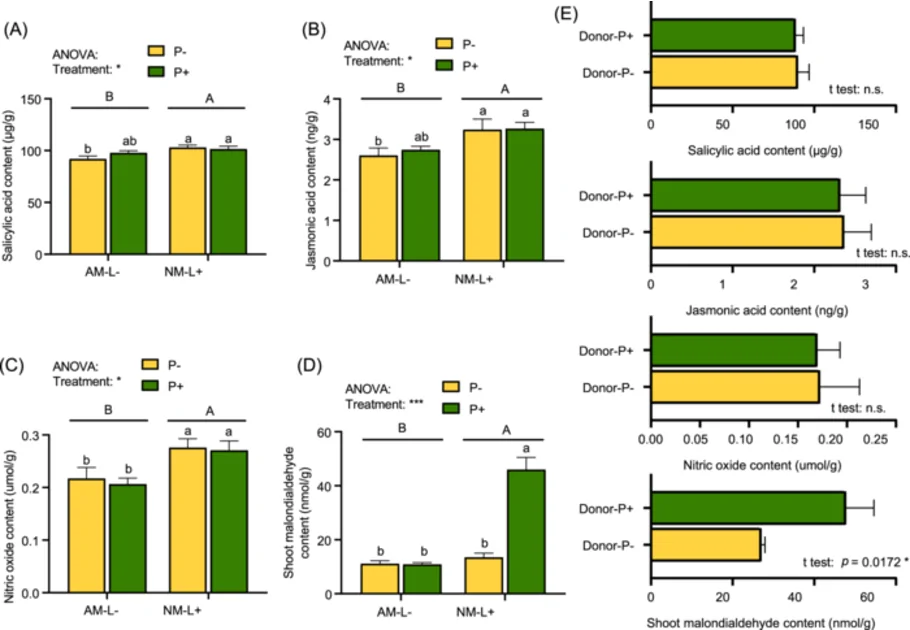
Plant hormone and malondialdehyde contents of the donor white clover (*Trifolium repens* L.) and receiver perennial ryegrass (*Lolium perenne* L.). (A) Salicylic acid, (B) jasmonic acid, (C) nitric oxide, and (D) malondialdehyde contents of perennial ryegrass linking donor white clover by common mycorrhizal networks (NM−L +) or not linked (AM−L−). (E) Salicylic acid, jasmonic acid, nitric oxide and malondialdehyde contents of the donor white clover. P+ , donor clover infected with the pathogen *Stemphylium sarciniforme*. P‐, donor clover not infected with the pathogen *S. sarciniforme*. Values are presented as the mean ± SEM of five replicates. Different lowercase letters above the bars indicate significant differences across the groups and treatments at *p* < 0.05 by LSD test. Different uppercase letters above the horizontal line indicate significant differences between the AM‐l‐ and NM‐L+ ryegrass at *p* < 0.05. Donor white clover data were analyzed using Student's *t*‐test. *p < 0.05. ***p < 0.001. n.s., no significant differences.

The contents of SA, JA, and NO in ryegrass were affected by the CMN connections (*p *< 0.001) (Figure 2A–C). The contents of SA, JA, and NO in the NM−L+ ryegrass was significantly higher by 7.79%, 21.58% and, 22.24%, respectively, compared to the AM−L− ryegrass (Figure 2A–C). In addition, the ryegrass linked through CMNs exhibited a higher content of malondialdehyde (MDA) when connected with the donor−P+ (P+ : the donor white clover was infected with pathogen) clover than when linked with donor−P− (P−: the donor white clover was not infected with pathogen) clover plants (Figure 2D). The content of MDA was significantly higher in the donor clover infected with the pathogen, whereas there were no significant differences in the contents of SA, JA, and NO compared to the uninfected clover plants (Figure 2E). Notably, there were no significant differences in the plant photosynthetic indicators across the different treatments for both donor clover and receiver ryegrass (Figure S2).

### Phyllosphere microbe diversity in clover and ryegrass leaves

**Figure 3:**
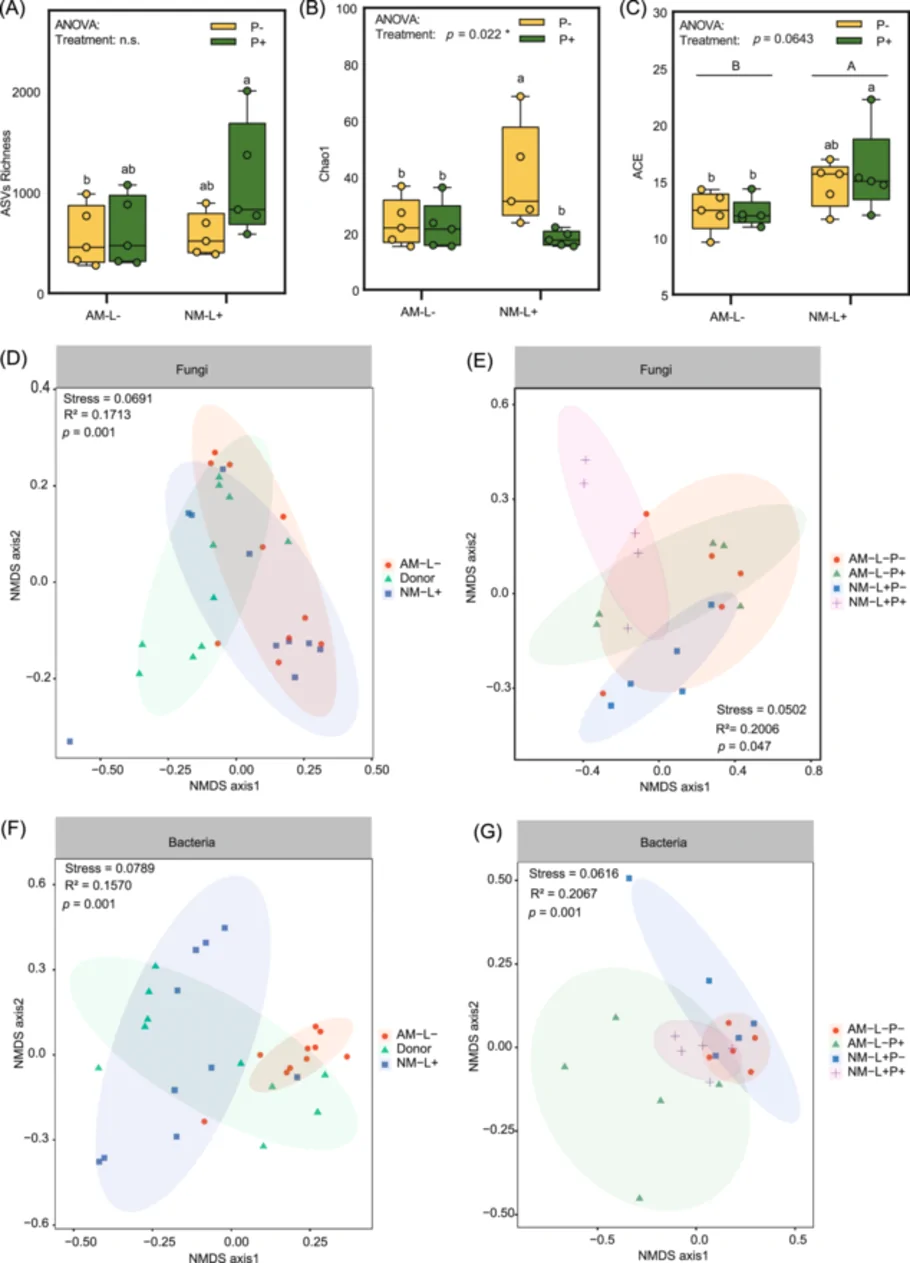
Phyllosphere fungi alpha diversity and phyllosphere microbial beta diversity. (A) Amplicon sequence variants (ASVs) richness, (B) chao1 index, and (C) abundance‐based coverage estimato (ACE) index of the fungi of perennial ryegrass (*Lolium perenne* L.) in the linking donor white clover (*Trifolium repens* L.) by common mycorrhizal networks (NM‐L +) or non‐linked (AM‐L‐). (D‐G) Nonmetric multidimensional scaling (NMDS) of the fungal (D, E) and bacterial (F, G) communities of perennial ryegrass (*L. perenne*) linked to the donor white clover (*T. repens*) by common mycorrhizal networks (NM‐L +) or not linked (AM‐L‐) and donor clover. P + , donor clover infected with the pathogen *Stemphylium sarciniforme*). P, donor clover not infected with the pathogen *S. sarciniforme*. Different uppercase and lowercase letters above the bars indicate significant differences across the groups and treatments at *p* < 0.05 by an LSD test. Different uppercases above the horizontal line indicate significant differences between the AM‐l‐ and NM‐L+ ryegrass at *p* < 0.05. *p < 0.05. n.s., no significant differences.

There were no significant differences in fungal alpha−diversity between the ryegrass treatments connected with donor−P− and donor−P+ clover (Figure S3). The NM−L+ ryegrass exhibited the highest fungal amplicon sequence variants (ASVs) richness when the donor clover was infected with the pathogen compared to the NM−L+P− and AM−L− ryegrass, but the difference was not significant (Figure 3A). Notably, the NM−L+P+ ryegrass had significantly higher fungal genus and species richness than the NM−L+P− and AM−L− ryegrass (Figure S4). The NML+P+ ryegrass had a significantly lower Chao1 index than the NML+P− ryegrass (Figure 3B). The fungal abundance‐based coverage estimato (ACE) index in the NM−L+ ryegrass was 24.60% higher than that in the AM−L− ryegrass (Figure 3C). Notably, there was no significant difference in the bacterial alpha−diversity among the receiver ryegrass (Figure S3). AM−L−P+ had a higher bacterial genus and species richness than the NM−L+P− and AM−L−P−ryegrass (Figure S5). The results suggests that the donor clover infected with pathogen changed the phyllosphere fungal diversity of the receiver ryegrass. However, the bacterial alpha−diversity indices, including Chao1, Shannon, Simpson, and Pielou, were lower in the donor−P+ clover compared to the donor−P− (Figure S6). The donor−P+ clover and donor−P− clover had similar levels of fungal diversity (Figure S7).

Regardless of the CMN connections, significant differences were observed in the fungal community structure between the clover and ryegrass leaves (NM−L+ vs Donor, *p* = 0.001; AM−L− vs Donor, *p *= 0.001) (Figure 3D, Table S1). The significant differences were observed in the fungal community structure between the NM−L+P− and NM−L+P+ ryegrass leaves (*p *= 0.017) (Figure 3E, Table S1). There were significant differences in the bacterial community structure in the clover and ryegrass leaves (NM−L+ vs Donor, *p* = 0.001; AM−L− vs Donor, *p *= 0.001) (Figure 3F, Table S2), and this difference was unaffected by whether or not the donor clover was infected with the pathogen (Figure S8 and Table S1,2). Notably, a significant difference was observed in the bacterial community structure between the AM−L− and NM−L+ groups (*p *= 0.003), regardless of whether the pathogen infects donor clover (Figure 3G, Table S2). Moreover, the community structure of the bacteria and fungi differed significantly between the clover leaves infected with the pathogen and uninfected clover leaves (fungi: *p* = 0.006; bacteria: *p *= 0.028), with some overlap observed between the two types of the fungal microbiome (Figure S9).

### Composition of the phyllosphere fungi and bacteria

The phyllosphere fungi in ryegrass groups were predominantly Ascomycota and Basidiomycota. The Ascomycota accounted for the highest proportion (58.22%) in the NM−L+P+ ryegrass, whereas it was the lowest (48.22%) in the NM−L+P− ryegrass (Figure S10). Olpidiomycota was the most abundant (2.90%) in the NM−L+P+ ryegrass, while it was relatively less abundant in the other three ryegrass groups (NM−L+P−, AM−L−P−, and AM−L−P+) (Figure S10). The endophytic fungi present in the ryegrass leaves were primarily categorized as unclassified Basidiomycota at the genus level, with insignificant differences in relative abundance observed among the four ryegrass groups (NM−L+P−, NM−L+P+, AM−L−P−, and AM−L−P +) (Figure S10). *Olpidium* was the most abundant (2.05%) in the NM−L+P+ ryegrass, with relatively lower abundance in the other three ryegrass groups (NM−L+P−, AM−L−P−, AM−L−P +). Notably, there was a higher abundance of *Olpidium* (1.10%) in the donor−P+ ryegrass compared to the donor P− (0.08%) ryegrass (Figure S10). The phyllosphere bacteria in the ryegrass were primarily composed of Proteobacteria. Proteobacteria was the most abundant (52.08%) in the AM−L−P− ryegrass, while it was the least abundant (29.09%) in the NM−L+P+ ryegrass (Figure S10). Desulfobacterota was the most abundant (1.33%) in the NM−L+P+ ryegrass and relatively less abundant in the other three ryegrass groups (NM−L+P−, AM−L−P−, and AM−L−P +) (Figure S10). Moreover, the phyllosphere bacteria *Bacteroides* were less abundant in the NM−L+P+ (1.06%) and AM−L−P+ (1.91%) ryegrass groups at the genus level compared to the NM−L+P− (0.43%) and AML−P− (0.68%) ryegrass groups. *Bacteroides* was less abundant (0.35%) in the donor−P+ ryegrass compared with the donor P− (1.13%) ryegrass (Figure S10). These results indicated that after infection of the donor clover with the pathogen, the phyllospheric microbial composition of the receiver ryegrass can be changed through CMNs.

### Shared microbes between the donor clover and the receiver AML− and NML+ ryegrass

**Figure 4:**
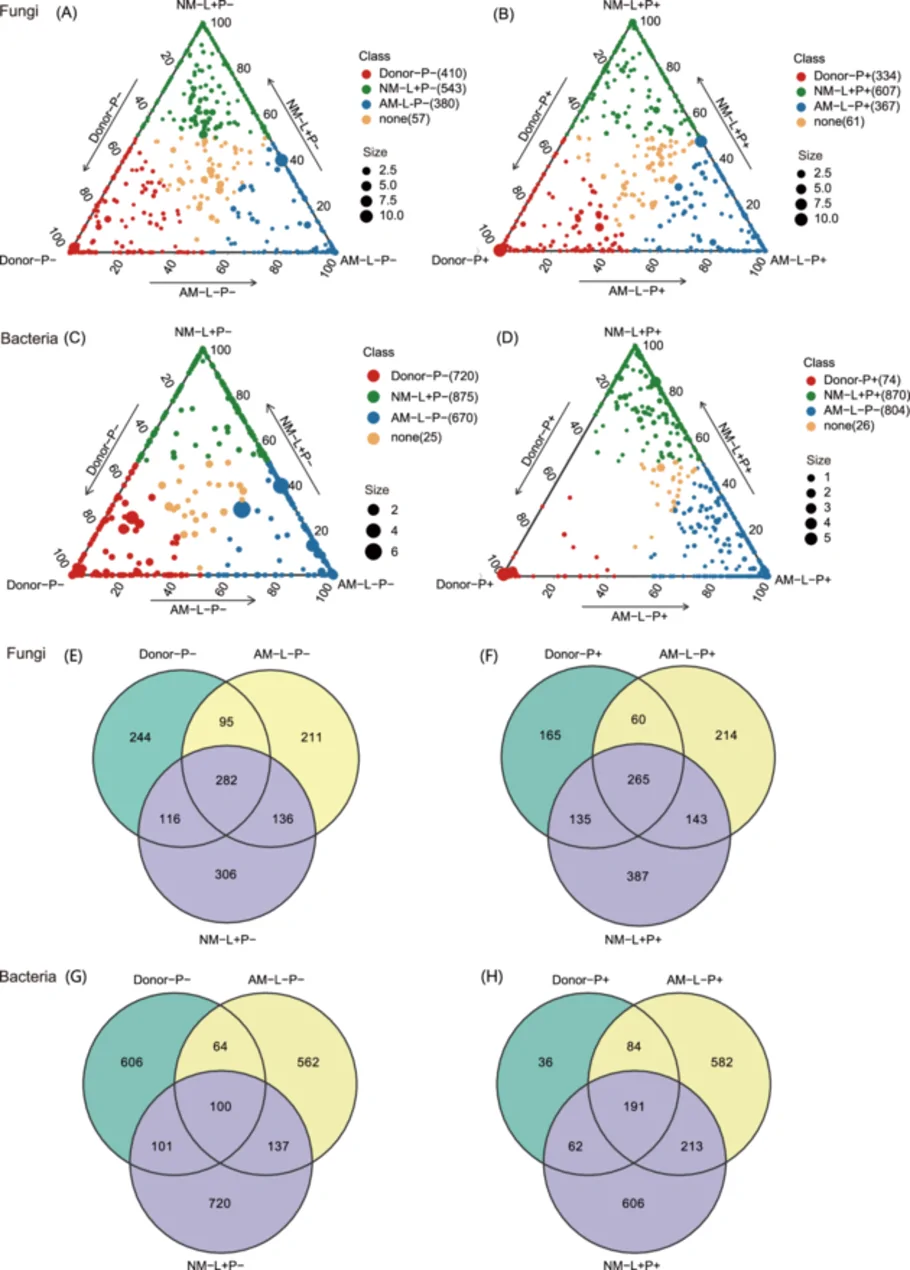
Fungal and bacterial ASVs shared among the donor clover, mycorrhizal network connections, and mycorrhizal network non‐connections in ryegrass. (A–D) Ternary plots for the fungal (A, B) and bacterial (C, D) ASVs. (E–H) Venn plots for the fungal (E, F) and bacterial (G, H) ASVs. Each symbol in the plot represents a single ASV, with colors indicating the compartment where it is primarily found. Red symbols, ASVs found >50% in the donor clover; green symbols, ASVs found >50% in the mycorrhizal‐linked ryegrass; blue symbol, ASVs found >50% in the non‐mycorrhizal‐linked ryegrass; orange symbols, general ASVs found in all compartments. The position of each symbol reflects the contribution of the indicated compartments to the total relative abundance.

There were slightly higher proportions of fungal ASVs in the NM−L+P+ ryegrass, whereas there were proportionately fewer fungal ASVs in the donor clover infected with the pathogen compared to the uninfected donor clover groups (Figure 4A,B). The prevalence of pathogen infection markedly decreased the proportion of shared bacterial ASVs in the donor clover (Figure 4C,D). The proportion of shared bacterial ASVs between NM−L+P− and NM−L+P+ remained consistent. Moreover, there were no significant differences in the shared fungal ASVs between donor−P+ versus NM−L+P+ (116) and donor−P+ versus AM−L−P+ (95). However, the shared fungal ASVs increased significantly in the donor−P+ versus NM−L+P+ (135) compared to the donor−P+ versus AM−L−P+ (60) (Figure 4E,F). The shared bacterial ASVs were markedly higher in the donor−P+ versus NM−L+P+ compared to the donor−P+ versus AM−L−P+ and increased from 64 to 101. However, when the pathogen infected the clover, the shared bacterial ASVs of the donor−P+ versus NM−L+P+ (62) were markedly reduced (Figure 4G,H). These results suggest that the phyllosphere microbiome of clover and ryegrass may communicate through CMNs.

### Linear discriminant analysis (LDA) effect size (LEfSe) analysis of the phyllosphere in the receiver ryegrass

**Figure 5:**
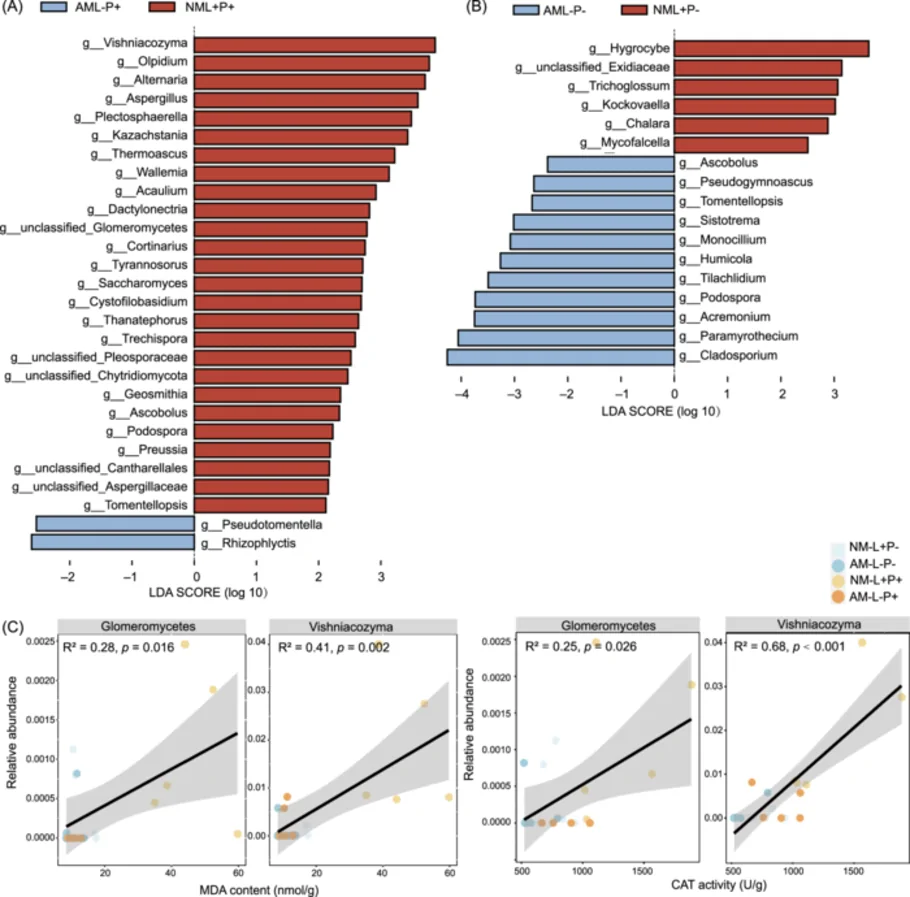
Linear discriminant analysis (LDA) effect size (LEfSe) analysis of the phyllosphere fungi in perennial ryegrass (*Lolium perenne* L.) and regression equations between the activity of catalase, content of malondialdehyde and genus and class levels of the ryegrass phyllosphere fungi. (A, B) LEfSe analysis of the phyllosphere fungi of perennial ryegrass linking donor white clover (*Trifolium repens* L.) by common mycorrhizal networks (NM‐L +) or not linked (AM‐L‐). P + , donor clover infected with the pathogen *Stemphylium sarciniforme*. P‐, donor clover not infected with the pathogen *S. sarciniforme*. The values are presented as the mean ± SEM of five replicates. The linear discriminant analysis (LDA) scores of the feature species drove the difference in the fungal and bacterial communities in the different treatments. Red columns, species that were more abundant in NM‐L+ ; blue columns, species that were more abundant in AM‐L‐. (C) Regression equations between the activity of catalase, content of malondialdehyde and genus and class levels of the ryegrass phyllosphere fungi. The coefficient of determination (R^2^) and statistical significance are shown.

In total, 28 fungal feature genera were identified. Only two fungal genera were more abundant (with higher LDA scores) in AM−L−P+, whereas the other 26 fungal genera were more abundant in the NML+P+ ryegrass. These included the *Vishniacozyma* genus and Glomeromycetes class, which may play a role in resistance to the pathogen (Figure 5A). Notably, NM−L+P+ was significantly more abundant than AM−L−P+. A total of 17 fungal feature genera were identified between AM−L−P− and NML+P− (Figure 5B). Moreover, 53 fungal feature genera were identified. The 39 fungal genera were more abundant in the NM−L+P+ ryegrass compared with NM−L+P−, including *Vishniacozyma* and *Glomus* (Figure S11). The activity of CAT and content of MDA significantly positively correlated with the relative abundance of members of the *Vishniacozyma* genus and the Glomeromycetes Class (Figure 5C). A total of 26 bacterial feature genera were identified, whereas the six genera were more abundant in the NM−L+P+ ryegrass compared with AM−L−P− (Figure S12). These results suggest that after infection of the donor clover with a pathogen, CMNs may drive the receiver ryegrass to have a stronger microbial response.

### Topological properties of the microbial networks

**Figure 6:**
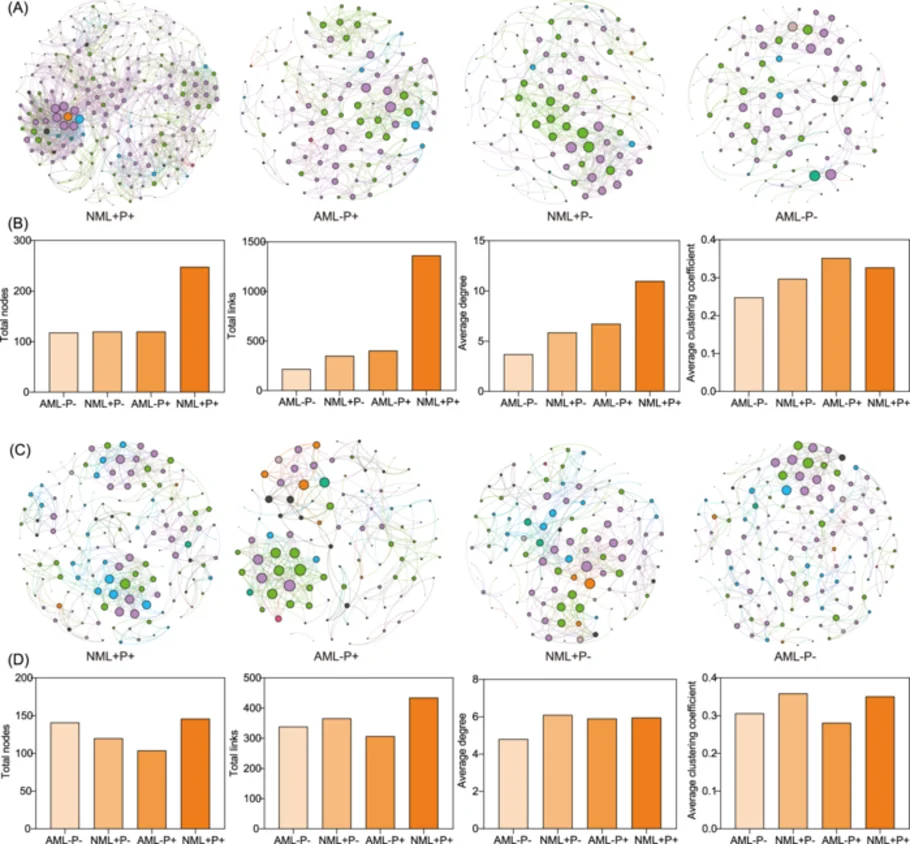
Co‐occurrence networks of the fungal and bacterial networks of perennial ryegrass (*Lolium perenne* L.) in the linking donor white clover (*Trifolium repens* L.) by common mycorrhizal networks (NM‐L+) or not linked (AM‐L‐). (A) Visualization of the fungal networks in the AM‐L‐ and NM‐L+ . (B) Topological properties of the fungal networks. (C) Visualization of the bacterial networks in the AM‐L‐ and NM‐L+ . (D) Topological properties of the bacterial networks. Different phyla are shown in different colors. P+ , donor clover infected with the pathogen *Stemphylium sarciniforme*. P−, donor clover not infected with the pathogen *S. sarciniforme*.

We constructed microbial networks to explore the potential microbial interactions within the different treatments. The microbial co−occurrence patterns varied markedly between the donor−P− and donor−P+ clover. In the donor−P+ clover, the total nodes, links and average degree of fungal and bacterial networks had higher values compared to the donor−P− clover (Figure S13). Similarly, the microbial co−occurrence patterns among NM−L+P+ and the other three treatments (AM−L−P+, NM−L+P−, and AM−L−P−) differed significantly as indicated by multiple topological properties of the networks. In the fungal networks, the total number of nodes and links were the highest at 248 and 1,363, respectively, in the NM−L+P+ ryegrass compared to the other three treatments (Figure 6A,B). Notably, the average degree of the network was the highest in NM−L+P+, suggesting that there was increased complexity within the fungal community following mycorrhizal connection and pathogen infection (Figure 6B). In the bacterial networks, the NM−L+P+ ryegrass had the highest number of links at 435 compared to the other three treatments (Figure 6C,D). Furthermore, the average clustering coefficient of the network was higher in the NM−L+ ryegrass compared to the AM−L− ryegrass, which indicated a more complex bacterial network in the mycorrhizal connection ryegrass (Figure 6D).

### Correlation analysis

**Figure 7:**
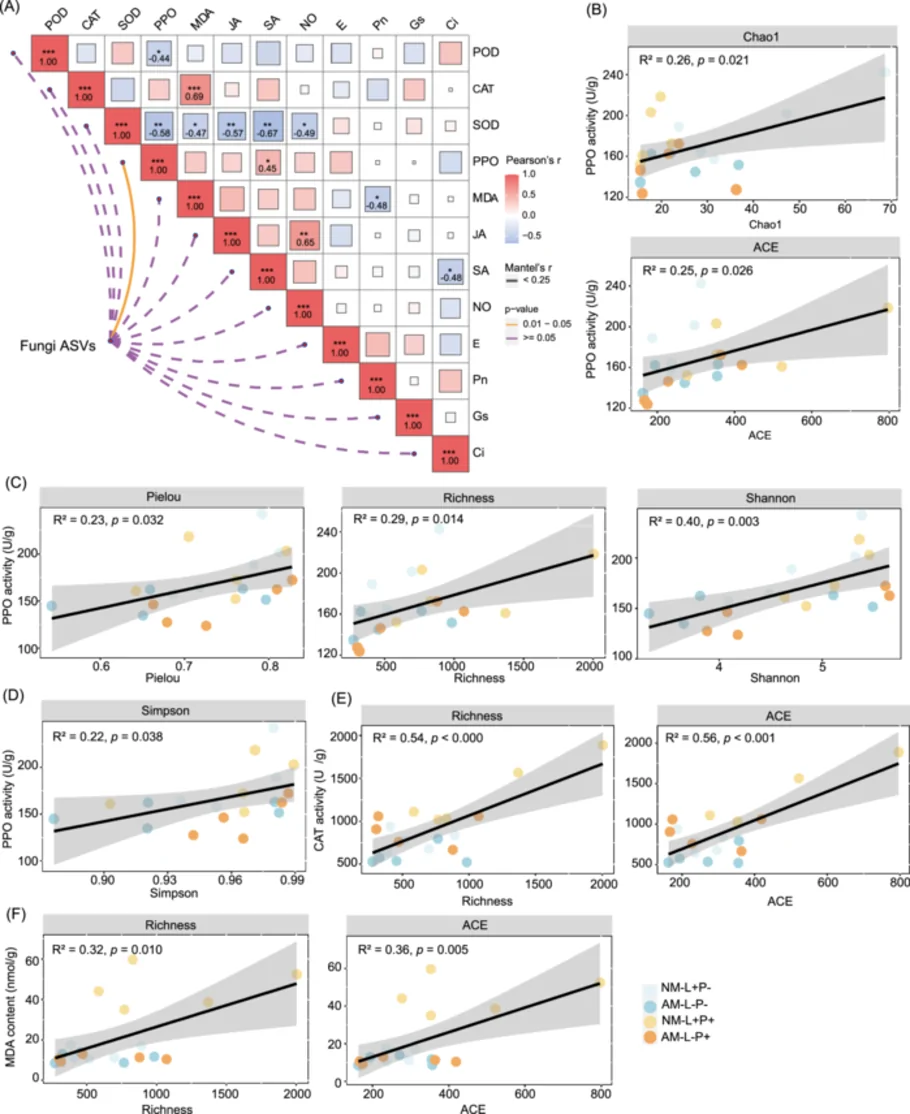
Correlation analysis of the physiological indicators and a mantel test analysis of the fungal ASV diversity and physiological indicators. (A) The mantel test analysis of the fungal ASV diversity and physiological indicators. (B–F) Regression equations between the fungal alpha‐diversity and the activities of polyphenol oxidase (B, C, D), catalase (E), and content of malondialdehyde (F). The coefficient of determination (*R* ^2^) and statistical significance are shown. CAT: catalase, PPO: polyphenol oxidase, MDA: malondialdehyde. *p < 0.05, **p < 0.01. ***p < 0.001.

A correlation analysis revealed significant positive correlations (*p* < 0.05) between CAT and MDA (*p* < 0.001), JA, and NO (*p* < 0.01), and SA and PPO (*p* < 0.05). The activity of SOD significantly negatively correlated with PPO, MDA, JA, SA, and NO (*p *< 0.05) (Figure 7A). A Mantel test analysis showed that PPO significantly correlated with fungal diversity (*p *< 0.05, mantel's *r* = 0.21) (Figure 7A). The activity of PPO significantly correlated with the fungal alpha−diversity indices, including the ASVs Richness and ACE, Chao1, Shannon, Simpson, and Pielou indices. Moreover, a significant positive correlation was observed between the activity of CAT, content of MDA, fungal ASVs Richness, and ACE index (Figure 7B–F). In addition, the activity of CAT significantly positively correlated with the bacterial Shannon index (Figure S14).

## 3 DISCUSSION

Under natural conditions, AM fungi can establish CMNs among various species of plants. However, previous research has primarily investigated the effects of pathogen infection on the phyllosphere microbiome of the same host plant, while there have been few studies that examined the influence of AM fungi on the plant response to pathogen infection at the plant community level. This is particularly true for the phyllosphere microbiomes. This study presents novel experimental evidence that pathogen infections can induce defense responses in neighboring plants between various plant species. Our findings revealed that ryegrass connected with clover through CMNs had higher levels of phyllosphere fungal diversity indices, including ACE, genus, and species richness. The infection of the donor white clover with pathogens changed the phyllosphere fungal community structure of the ryegrass connected with clover through the CMNs. In addition, there were differences in the bacterial community structure between the ryegrass interconnected by the CMNs and ryegrass without the CMNs. These observations indicated that the ryegrass connected with clover through CMNs had the most complex fungal microbial network structure, which could potentially enhance its resistance against the pathogen. Furthermore, the CMNs altered the phyllosphere microbiome in the receiver plants, which correlated with their ability to induce defense mechanisms, such as the increase in CAT activity in the donor clover and receiver ryegrass. These findings showed that the CMNs play a vital role in shaping the defense response at the plant community level.

Mycorrhizal colonization was observed in the ryegrass of NM−L+, which indicated the formation of CMNs by AM fungal hyphae between the donor clover and receiver ryegrass. Our experimental design minimized the potential effects of soil diffusion or root−to−root contact and primarily focused on the effects of mycelial connections between plants. The 5 cm PVC tubes formed a buffer zone and prevented direct contact between the roots of white clover and ryegrass with a 0.45 and 37 µm nylon mesh, respectively (Figure 1F). Nevertheless, it is important to acknowledge that under different natural conditions, alternative pathways besides AM fungal mycelia in the rhizosphere could serve as signal conduits. For example, the signal transfer between plants might occur through a liquid stream or film layer, and the presence of the fungal mycelia could potentially enhance the formation of these pathways [35]. The meshes used in our experiment were water permeable, thus, it is unlikely that our method of preventing hyphal connections (0.45 µm mesh core) also inhibited the formation of liquid streams or film layers. Consequently, the lead root exudates might mediate plant−plant communication between plants of different species. If the root exudates mediate inter−plant communication, the induced defense responses could be detected in ryegrass that was not associated with the CMNs (AM−L−). However, we observed higher activities of CAT and PPO and higher levels of JA, SA, and NO in NM−L+ than in AM−L− ryegrass. This finding indicated that the induced defense responses did not occur when ryegrass was not connected to clover through the CMNs, which indirectly demonstrated that the CMNs can prompt neighboring plants to generate defense responses at the community level within ecosystems. Based on our experimental design and results, we attribute the signal transfer to the AM fungal mycelia or potentially to some physical phenomenon associated with the CMNs [12].

Pathogen infection will reduce the photosynthesis of plants and affect their growth. However, AM fungi tend to provide mineral nutrients to those plants that can best provide photosynthetic C through CMNs to obtain more carbon rewards from the plant community [36, 37, 38]. In this study, although the clover was infected by pathogens, the photosynthesis of the diseased clover occurred at a rate comparable to that of the healthy clover. Importantly, owing to species differences, the net photosynthetic rate of the white clover is significantly higher than that of ryegrass. In addition, the ryegrass linked to clover infected with pathogen through the CMNs had the lowest net photosynthetic rate. However, the difference was not significant. This may be caused by the transfer of more nutrients to the clover by the AM fungi through CMNs.

### CMNs changed the activities of defensive enzymes and the contents of hormones in the receiver ryegrass

The findings of this study demonstrate that the CMNs play a vital role in determining the response of receiver plants connected to the donors infected with pathogens. Elevated activities of CAT were observed in the donor clover infected with the pathogen and the receiver ryegrass connected through CMNs (Figure 1). This result suggests that the CMNs may transfer profiles related to defense enzymes at the plant community levels and possibly enhance the resistance of plants in ecosystems. Furthermore, significantly increased levels of the activities of PPO and CAT and the contents of MDA were observed in ryegrass connected through the CMNs. Previous studies have shown that the CMNs can transfer disease and pest defense signals to nearby plants of the same species [11, 12]. This study provides novel evidence that the signaling molecules produced by pathogens can be transferred from infected plants to uninfected plants of different species via CMNs. These findings support our hypothesis that ryegrass linked to pathogen−infected white clover by CMNs exhibits defense responses similar to those of infected donor plants, which induces changes in the levels of defense compounds.

Various plant hormones, including auxins, SA, and JA, mediate plant defense against pathogens [39]. NO is an important regulatory component in the signaling pathways, which is implicated in plant defense responses [40]. This study revealed that there were significantly higher levels of SA, JA, and NO in ryegrass linked with clover through the CMNs. This phenomenon could be attributed to the sharing of defense response molecules between clover and ryegrass through CMNs, thereby improving the overall health of the system. These changes may not necessarily depend on whether the pathogen infects the donor plant. Importantly, in this study, some communication might have occurred between the donor and receiver plants of different species since the activities of defensive enzymes increased in both the donor and receiver plants linked by the CMNs. After establishment of the CMNs with the AM fungus *Glomus mosseae* between tomato, inoculation of the donor plants with the pathogen *Alternaria solani* led to increases in the disease resistance and activities of the putative defensive enzymes, POD, PPO, chitinase, ß−1,3−glucanase, phenylalanine ammonia−lyase and lipoxygenase in healthy neighboring receiver plants [11]. These indicate that the plant defense enzymes may be important signal response compounds mediated by CMNs.

Importantly, the signaling compounds that transferred between plants through these CMNs have not yet been identified. Johnson and Gilbert (2015) pointed out that the signaling compounds may be molecules that are already known to be transported in AM fungal hyphae [13], including lipids, such as triacylglycerols [41], phosphate transporters and amino acids [42], or on compounds known to elicit or be produced by established plant–mycorrhizal fungus signaling pathways [43]. However, there is no direct evidence for this hypothesis to date. In addition, the signaling molecules may be specific to the types of AM fungi, hosts, or pathogen. Although there is little knowledge of the signaling compounds transferred via the CMNs eliciting defensive response in uninfected plants, it is an important target for future research to identify the compounds transported by CMNs and elucidate the mechanisms that regulate the signal transport through CMNs that is induced by pathogens.

### CMNs altered the diversity and community structure of the phyllosphere microbes, which enhanced the microbial cooperation and network complexity

The plant−associated microbial community (microbiome) is important in facilitating plant−plant communications [6]. Infection by pathogens can change the diversity and community structure of the plant phyllosphere microbes [27]. However, only a few studies have explored the impact of AM fungi on the phyllosphere microbiome. In addition, pathogen infection can change the diversity and community structure of the plant phyllosphere microbiome [27, 44]. Owing to a shift in the phyllosphere microbiome of citrus upon *D. citri* infection, there was significantly higher richness of epiphytic bacteria in the infected leaves than in the uninfected leaves [27]. However, this only indicated that pathogen infection can alter the diversity of phyllosphere microbiome in individual plants. Importantly, to our knowledge, no studies have evaluated the effects of CMNs on the receiver phyllosphere microbiome of receiver plants when the donor plant is infected with a pathogen. Thus, this study is the first to demonstrate that CMNs can influence the diversity and community structure of the phyllosphere microbiome in receiver plants. The results show that infection of the donor clover with the pathogen changed the phyllosphere fungal diversity and community structure of the receiver ryegrass. The phyllosphere bacterial community structure of the receiver ryegrass changed significantly with the CMNs connection, but it was not affected by infection in the donor white clover by the pathogen. The differences observed in the alpha− and beta−diversity of the phyllosphere microbes, particularly in the fungal community, suggest that the CMN connections can influence the microbiota and potentially play a role in the plant's defense against pathogens.

The alteration of the receiver plant phyllosphere microbiome induced by CMNs could be attributed to several factors. First, certain bacteria, such as phosphate−solubilizing bacteria and rhizobia, have flagellar motions that enable them to move through the water film on the hyphal surface [45, 46, 47]. Secondly, plant roots and the rhizosphere serve as a reservoir for microbes that are translocated to the aboveground parts of the plant, including the phyllosphere [31]. Previous studies demonstrated that the AM fungi modulate the microbiome and selectively assemble their microbial communities from the surrounding soil in the plant rhizosphere [29] and hyphosphere [30, 48]. In this study, the number of shared fungal ASVs between the donor and ryegrass linked by the CMNs significantly increased after the donor clover was infected with the pathogen (Figure 4). A plausible explanation for this observation is the formation of a unique rhizosphere and hyphosphere microbiome following the infection of the donor plant with the pathogen. This might have facilitated the migration of bacteria to the rhizosphere and hyphosphere of the receiver plant through the hyphae, thereby changing the phyllosphere microbiome of the receiver plant.

Microbial cooperation and network complexity can impact plant health [49]. In this study, the donor clover infected with the pathogen exhibited a more complex fungal and bacterial network structure compared to the uninfected clover. Notably, as shown in Figure 6, a significantly more intricate fungal network structure was observed in ryegrass connected to the clover infected with the pathogen than in both the non−CMNs and ryegrass connected to healthy clover. Therefore, the microbial network complexity increased after the clover was infected with the pathogen, which subsequently affected the adjacent ryegrass through the CMNs. This ultimately enhanced the complexity of the ryegrass microbial network and improved the stability of the system. The microbial networks of CMNs were more robust, which supported the principle that greater complexity enhances the stability of ecosystems [50]. Phyllosphere microbes and pathogens share similar ecological niches and compete for nutrients and space within the confined leaf environments; this could potentially inhibit infection with pathogens [27, 51]. In this study, the increase in microbial network complexity may correlate with a significant increase in the shared fungal ASVs between the donor and ryegrass linked by the CMNs under pathogen infection. Therefore, this validated our hypothesis regarding the alteration of the phyllosphere microbial diversity and community structure by the CMNs, which thereby induced defense responses against pathogens in the receiver plants.

### CMNs modify the phyllosphere microbes, which induces and enhances the plant resistance against pathogens

Our subsequent analysis revealed that the CMNs influence the diversity and composition of phyllosphere microbes in receiver plants, which correlates with the defense signaling substances induced by the CMNs. A Mantel test analysis showed a significant positive correlation between fungal ASV abundance and PPO. A correlation analysis showed that the activities of PPO and CAT were positively associated with the fungal alpha−diversity indices. These results indicate that the changes in plant defense mechanisms in the receiver plants could be another pathway through which CMNs affect the phyllosphere microbes of neighboring plants. Studies have demonstrated the crucial rules of CAT and PPO in plant growth, development and defense responses [52, 53]. AM fungi increase the activities of defense enzymes, such as CAT, PPO, and POD, in plants during pathogen infection, thereby enhancing the plant resistance to pathogens [54, 55]. Previous studies have reported that plants recruit beneficial microbes to enhance their defensive capabilities against pathogens [26, 27], possibly through the induction of plant metabolic defense mechanisms by phyllosphere microbes [56]. The correlation between the activities of CAT and PPO and the fungal alpha−diversity suggests that the microbial shifts are directly related to the activation of defense enzymes.

Furthermore, an LEfSe analysis was conducted to identify feature genera across the different treatments, particularly those that exhibited significantly higher abundance in the NM−L+P+ ryegrass compared to the AML−P+. In this study, 26 genera were more abundant in the NML+P+ ryegrass, including *Vishniacozyma*. *Vishniacozyma* genus fungi can against various plant pathogens, including pathogens *Fusarium* [57], *Moniliophthora roreri* [58], *Botrytis cinerea* [59], *Penicillium expansum*, and *Cladosporium* spp. [60]. Therefore, we hypothesized that fungi of the *Vishniacozyma* possess broad pathogen resistance. Particularly in this study, the *Vishniacozyma* genus was significantly enriched in the ryegrass linked by diseased clover through the CNMs, which indicated that it may play an crucial role in white clover−ryegrass systemic disease resistance. This study revealed that the activity of CAT and content of MDA significantly positively correlated with the relative abundance of these endophytes. This indicates that the induction of phyllosphere microbes could play an important role in regulating the defense responses against pathogens mediated by CMNs. However, further study is required to elucidate its specific interaction with the antioxidant enzymes. Furthermore, the increased microbial enrichment may contribute to the elevated content of malondialdehyde observed in ryegrass linked with clover infected with the pathogen through the CMNs [28].

## 4 CONCLUSIONS

This study demonstrates that the CMNs provide a channel for interplant communication, which enables plants of different species to prepare for pathogen infections without direct exposure. In summary, CMNs change the microbial abundance and community structure of the neighboring plants following pathogen infection in the donor plants, which further enhances microbial interaction and network complexity in neighboring plants. This activation may trigger defense responses in the receiver plants against potential pathogens, which enhances the system's ability to resist infections. Further studies should be conducted to explore whether these changes mediated by CMNs increase the resistance of the interconnected plant communities and whether these phenomena occur in natural settings.

## 5 METHODS

### Plants and fungi

White clover (*Trifolium repens* L.) and perennial ryegrass (*Lolium perenne* L.) were used as the experimental materials in this study. *Funneliformis mosseae* (No. BGCNM02A) was used as the AM fungus. The AM fungal inoculum consisted of dry soil, AM fungal spores (>50 spores per g soil), mycelium, and colonized white clover root fragments, and it was prepared from pots of white clover grown in a greenhouse. The leaf spot pathogen *Stemphylium sarciniforme* was isolated from diseased red clover (*Trifolium pratense* L.) in fields in Minxian, Gansu, China. The pathogen was identified through morphological evaluation and molecular techniques (GenBank accession numbers: PP212851 and PP236998). Importantly, the pathogen *S. sarciniforme* is an important leaf spot pathogen of species of *Trifolium*, including white clover [61].

### Experimental design

The experimental design was adapted as described by Babikova et al. (2013) [12]. Two distinct underground connections were established between the donor plant (white clover) and the receiver plant (perennial ryegrass) in a growth chamber that measured 40 × 30 × 27 cm (Figure 1F). The donor plants were planted in the middle of the growth chamber, inoculated with the AM fungus, and either infected (P+) or not infected with the pathogen (P−). Two pots that contained receiver plants were positioned on either side of the growth chamber. The CMNs of the AM fungal mycelia between the donor and receiver plants were either linked (L+) or not linked (L−) using a 5 cm length polyvinyl chloride (PVC) tube with a diameter. The growth medium was added in a 5 cm PVC tube. There were two treatments. (1) In the first treatment, a nylon mesh (37 µm) was placed on both sides of the PCV tube of 5 cm. This enabled the AM fungal mycelia from the donor plant to pass through and form CMNs. The receiver plant was not inoculated with AM fungi (NM−L+). (2) In the second treatment, a nylon mesh (0.45 µm) was placed on both sides of the PCV tube, and it prevented the AM fungal mycelia from passing through. Thus, this plant could not form CMNs. The receiver plant was inoculated with the AM fungus (AM−L−) in this group. Importantly, the 5 cm PVC pipe prevented direct contact between the roots of white clover and ryegrass (Figure 1F). Ten mesocosms were established in a greenhouse, with five that were white clover infected with the pathogen. The other five were uninfected clover. There were five replicates in this experiment, which was conducted in the College of Pasture Agriculture Science and Technology, Yuzhong campus of Lanzhou University, Yuzhong, China.

### Growth medium

The soil mixture used in the experiments was prepared using Semi−Luvisol soil obtained from the Xinlong Mountains in northwest China [62]. The mix was composed of 30% soil and 70% sand. The two components were passed through a 2 mm sieve and then sterilized by autoclaving twice for 1 h each at 121°C over 3 days and dried for 36 h at 110°C. There was 17.66 mg·kg^−1^ of total P in the soil mix, which had a pH of 7.3.

The receiver plants were grown in 17.6 cm high non−draining plastic pots that were 11 cm in diameter and filled with 1.4 kg of soil mix. The AM treatment in the donor and receiver plants utilized 100 g of AM fungal inoculum that was evenly mixed into the soil in each pot. In contrast, the pots designated for the non−inoculated treatment received 100 g of sterilized AM fungal inoculum, and solutions that lacked the AM fungus that contained other microbes and were previously prepared microbial filtrates (50 μm) per pot [62].

### Greenhouse experiments

White clover and perennial ryegrass seeds were obtained from the Forage Seed Testing Centre, Ministry of Agriculture and Rural Ministry of China, Lanzhou, China. The seeds were surface−sterilized by immersion in 1% NaClO for 10 min and then rinsed three times with sterile water. The seeds were then soaked in 75% ethanol for 3 min and rinsed three times with sterile water. The seeds were cultured for germination on moist filter paper in the dark at 25°C. Five ryegrass plants were planted in each pot, and 14 white clover plants were planted in each pot.

The white clovers were inoculated with the pathogen *S. sarciniforme* 10 weeks after planting. The fungus was grown on potato dextrose agar (PDA), and its conidia were harvested to prepare a pathogen inoculum that contained 6 × 10^6^ conidia/mL [36]. At 40 days after emergence, 10 pots of white clover were each sprayed with a 20 mL suspension that contained 6 × 10^6^ conidia/mL of *S. sarciniforme* and were considered the pathogen treatments. The remaining 10 pots of white clover were sprayed with 20 mL of sterile water to generate the control treatments. After the plants were sprayed with the pathogen or sterile water, they were covered with black plastic cages for 48 h to retain moisture to facilitate infection by the pathogen. The symptoms of white clover anthracnose were observed daily by monitoring the plants. Typical disease symptoms were observed 7 days after inoculation (Figure S15). The infection phase of the experiment lasted for another 7 days, and the plants were then harvested. All the donor and receiver plants were enclosed in polyethylene terephthalate tubes to prevent plant−to−plant communication by aerial volatiles.

### Plant harvest and analysis of the antioxidant parameters

At 7 days after pathogen inoculation, the photosynthetic parameters were measured, including the net photosynthetic rate (Pn), intercellular carbon dioxide concentration (Ci), transpiration rate (E), and stomatal conductance (Gs), as described in more detail in Methods S1. Fresh leaves of the white clover and the perennial ryegrass plants were harvested from each plant in the pot. A total of 1 g of leaves were used to determine the activity of antioxidant enzymes, including POD, SOD, CAT, and PPO. Approximately 0.8 g of the leaves were used to determine the concentrations of SA, JA, NO, and MDA. In addition, approximately 1 g of leaf samples were shaken and surface sterilized to extract DNA and sequence the amplicons as described in Methods S2 [28, 63].

The remaining samples were cut and placed into envelope bags that were dried and weighed. The samples were incubated at 115°C for 30 min and dried at 65°C for 48 h until a constant weight was achieved. The content of water in the samples was then calculated, and the total dry weight of the stems and leaves was determined. The roots were harvested from each growth chamber and washed with water. Approximately 0.5 g of fine roots were selected and cut to determine the rate of mycorrhizal colonization [64]. The other portion of roots was also placed in dried and weighed envelope bags and then dried at 65°C for 48 h until a constant weight was achieved. The content of water in the roots was calculated, and the total dry weight of the roots was determined. The activities of POD, SOD, and PPO and the content of MDA were determined using colorimetric assays with kits (Sangon Biotech, Shanghai, China) as described by Gao et al. [65]. The contents of SA, JA, and NO were evaluated by an enzyme−linked immunosorbent assay (ELISA) [62].

### DNA extraction and amplicon sequencing

The DNA was extracted using a TGuide S96 Magnetic DNA Kit (Tiangen Biotech Co., Ltd. Beijing, China) according to the manufacturer's instructions. The amount of DNA in the samples was measured with a Qubit dsDNA HS Assay Kit and a Qubit 4.0 Fluorometer (Invitrogen, Thermo Fisher Scientific, Waltham, MA, USA). The bacterial 16S ribosomal RNA (rRNA) gene V3–V4 region was amplified using primers 335 F (5′−CADACTCCTACGGGAGGC−3′) and 769 R (5′−ATCCTGTTTGMTMCCCVCRC−3′). The fungal ITS1 gene was amplified using primers ITS1F (5′−CTTGGTCATTTA GAGGAAGTAA−3′) and ITS2 (5′−GCTGCGTTCTTCATCGATGC−3′) [66] as described in Methods S3.

An Illumina NovaSeq. 6000 platform (Illumina, San Diego CA, USA) was used to sequence the constructed library. The raw data were filtered using Trimmomatic (v. 0.33) to ensure high−quality sequence data [67]. The primer sequences were identified and removed using Cutadapt (v. 1.9.1) [68]. The paired−end (PE) reads obtained from the previous steps were assembled by USEARCH (v. 10), and the chimeras were removed using UCHIME (v. 8.1). The high−quality reads generated from the previous steps were used for subsequent analyses. The clean reads then were subjected to feature classification to output the ASVs (amplicon sequence variants) by DADA2 [68], and the ASVs with a reabundance <0.005% were filtered. The clean fungal reads per sample ranged between 285,082 and 390,488 in ryegrass and 403,254 in clover. The number of clean bacterial reads per sample ranged between 72,234 and 79,259 in ryegrass and 73,693 in white clover. The ASVs were obtained from the phyllosphere fungi and bacteria, with 3,111 and 6,472 identified in ryegrass and 1,595 and 1,701 identified in clover, respectively (Table S3,4). These analyses and the dilution curves indicate that the sequencing depth met the standard for the data to be considered high quality (Figure S16).

### Statistical analysis

The receiver ryegrass data were subjected to an analysis of variance (ANOVA) using the R statistical program (v. 4.2.0). Significant differences among the treatments were determined by LSD tests (*p* < 0.05). The donor white clover data were analyzed using a Student's *t−*test. The taxonomy of the ASVs was annotated using the Naive Bayes classifier in QIIME2 [69] in the SILVA database [70] (release 132) with a confidence threshold of 70%. The degree of alpha−diversity was calculated and visualized using the QIIME2 and R software, respectively. The beta−diversity was determined to evaluate the similarity of microbial communities from different samples using QIIME. Nonmetric multidimensional scaling (NMDS) was used to analyze the beta−diversity. PERMANOVA was then used to assess the Bray−Curtis distances of the ASVs of clover and ryegrass using the “vegan” package in R. Furthermore, we used LEfSe analysis to determine the significant taxonomic differences among the groups [71]. A logarithmic LDA score of 2.0 was set as the threshold for discriminative features. A Mantel test was used to analyze the impact of the diversity of ASVs on enzyme activity, plant hormones, and photosynthetic indicators. The regression analyses were calculated using the “ggpmisc” package and visualized by the “ggplot2” package in R. Microbial co−occurrence networks were independently created for each treatment using ASVs based on the parameter that the frequency of ASV appearing in the sample ≥3/5. Robust correlations based on Spearman's correlation coefficients $r_{s}$ of >0.8 or <0.8 and false discovery rate−corrected *p* values <0.05 were used to construct the networks [52]. We calculated the characterization of the networks to estimate their complexity, including the total number of nodes, number of links, average clustering coefficient, and average degree. Here, the average clustering coefficient referred to the degree to which the nodes tended to cluster together. The average links per node were defined by the average edges of each node. These characterization values were calculated using the “igraph” package [49]. The networks were visualized using interactive Gephi software (v. 0.9.7).

## AUTHOR CONTRIBUTIONS

Zhibiao Nan, Tingyu Duan, and Yong Wei planned and designed the research; Tingyu Duan, Yong Wei, Yingde Li, and Youlei Shen performed the research; Tingyu Duan, Yingde Li, and Rongchun Zheng, and Yajie Wang analyzed the data, while Tingyu Duan, Yingde Li, and Yong Wei wrote the manuscript. All authors have read the final manuscript and approved it for publication.

## CONFLICT OF INTEREST STATEMENT

The authors declare no conflicts of interest.

## 1 ETHICS STATEMENT

No animals or humans were involved in this study.

## Supporting information


**Figure S1:** Biomass and mycorrhizal colonization of white clover (*Trifolium repens*) and perennial ryegrass (*Lolium perenne*). **Figure S2:** Photosynthesis indices of white clover (*Trifolium repens*) and perennial ryegrass (*Lolium perenne*). **Figure S3:** The bacteria alpha diversity of perennial ryegrass (*Lolium perenne*). **Figure S4:** Fungi diversity of perennial ryegrass (*Lolium perenne*). **Figure S5:** Bacteria diversity of perennial ryegrass (*Lolium perenne*). **Figure S6:** The fungi alpha diversity of donor white clover (*Trifolium repens*). **Figure S7:** The bacteria alpha diversity of donor white clover (*Trifolium repens*). **Figure S8:** The beta diversity of perennial ryegrass (*Lolium perenne*). **Figure S9:** The beta diversity of donor white clover (*Trifolium repens*). **Figure S10:** Composition of the phyllosphere fungi and bacteria in perennial ryegrass (*Lolium perenne*) and white clover (*Trifolium repens*). **Figure S11:** LEfSe analysis of phyllosphere fungi of perennial ryegrass (*Lolium perenne*) linking donor white clover (*Trifolium repens*) by common mycorrhizal networks (NM‐L+) or un‐linking (AM‐L‐) in genus level. **Figure S12:** LEfSe analysis of phyllosphere bacteria of perennial ryegrass (*Lolium perenne*) linking donor white clover (*Trifolium repens*) by common mycorrhizal networks (NM‐L+) or un‐linking (AM‐L‐) in genus level. **Figure S13:** Co‐occurrence networks of donor white clover (*Trifolium repens*). **Figure S14:** Regression equations between catalase activity and bacteria alpha diversity of receiver perennial ryegrass (*Lolium perenne*). **Figure S15:** The pictures of diseased leaves of donor white clover (*Trifolium repens*) mycorrhizal colonization. **Figure S16:** Dilution curves of the phyllosphere fungi and bacteria in white clover (*Trifolium repens*) and perennial ryegrass (*Lolium perenne*).

**Table S1:** Permutational multivariate two‐way analysis of variance (PERMANOVA) table of the phyllosphere fungi of receiver perennial ryegrass (*Lolium perenne*) and donor white clover (*Trifolium repens*). **Table S2:** Permutational multivariate two‐way analysis of variance (PERMANOVA) table of the phyllosphere bacteria of the receiver perennial ryegrass (*Lolium perenne*) and the donor white clover (*Trifolium repens*). **Table S3:** Summary statistics for the fungal sequences from perennial ryegrass (*Lolium perenne*) in linking to the donor white clover (*Trifolium repens*) by common mycorrhizal networks (NM‐L+) or not linking (AM‐L‐) and donor clover. **Table S4:** Summary statistics for the bacterial sequences from perennial ryegrass (*Lolium perenne*) in linking the donor white clover (*Trifolium repens*) by common mycorrhizal networks (NM‐L+) or not linking (AM‐L‐) and donor clover.

## Data Availability

Raw sequence data can be accessed at the NCBI BioProject database https://www.ncbi.nlm.nih.gov/ (BioProject ID: PRJNA1113835 and PRJNA1113852). The data and scripts used are saved in GitHub https://github.com/talent423/IMO-2024-0085.git/. Supplementary materials (methods, figures, tables, graphical abstract, slides, videos, Chinese translated version, and update materials) may be found in the online DOI or iMeta Science http://www.imeta.science/imetaomics/.
