# Supplementary material for: Common mycorrhizal networks enhance defense responses against pathogens in neighboring plants
Source: IMetaOmics. 2024 Dec 7;2(1):e46. doi: 10.1002/imo2.46 (PMC12806432; doi:10.1002/imo2.46)
Supplement: Supplementary file 1 — Figure S1: Biomass and mycorrhizal colonization of white clover (Trifolium repens) and perennial ryegrass (Lolium perenne). Figure S2: Photosynthesis indices of white clover (Trifolium repens) and perennial ryegrass (Lolium perenne). Figure S3: The bacteria alpha diversity of perennial ryegrass (Lolium perenne). Figure S4: Fungi diversity of perennial ryegrass (Lolium perenne). Figure S5: Bacteria diversity of perennial ryegrass (Lolium perenne). Figure S6: The fungi alpha diversity of donor white clover (Trifolium repens). Figure S7: The bacteria alpha diversity of donor white clover (Trifolium repens). Figure S8: The beta diversity of perennial ryegrass (Lolium perenne). Figure S9: The beta diversity of donor white clover (Trifolium repens). Figure S10: Composition of the phyllosphere fungi and bacteria in perennial ryegrass (Lolium perenne) and white clover (Trifolium repens). Figure S11: LEfSe analysis of phyllosphere fungi of perennial ryegrass (Lolium perenne) linking donor white clover (Trifolium repens) by common mycorrhizal networks (NM‐L+) or un‐linking (AM‐L‐) in genus level. Figure S12: LEfSe analysis of phyllosphere bacteria of perennial ryegrass (Lolium perenne) linking donor white clover (Trifolium repens) by common mycorrhizal networks (NM‐L+) or un‐linking (AM‐L‐) in genus level. Figure S13: Co‐occurrence networks of donor white clover (Trifolium repens). Figure S14: Regression equations between catalase activity and bacteria alpha diversity of receiver perennial ryegrass (Lolium perenne). Figure S15: The pictures of diseased leaves of donor white clover (Trifolium repens) mycorrhizal colonization. Figure S16: Dilution curves of the phyllosphere fungi and bacteria in white clover (Trifolium repens) and perennial ryegrass (Lolium perenne). [file IMO2-2-e46-s001.doc]

**Supporting information to**

**Common mycorrhizal networks enhance defense responses against pathogens in neighboring plants**

**Running title:** Common mycorrhizal networks enhance neighboring plant defense

Yingde Li^1,2,3,4#^, Yong Wei^1,2,3,4#^, Youlei Shen^1,2,3,4^, Rongchun Zheng^1,2,3,4^, Yajie Wang^1,2,3,4^, Tingyu Duan^1,2,3,4*^, Zhibiao Nan^1,2,3,4*^

^1^State Key Laboratory of Herbage Improvement and Grassland Agro-ecosystems, Lanzhou University, Lanzhou 730000, China

^2^Key Laboratory of Grassland Livestock Industry Innovation, Ministry of Agriculture and Rural Affairs, Lanzhou 730000, China

^3^Engineering Research Center of Grassland Industry, Ministry of Education, Gansu Tech Innovation Centre of Western China Grassland Industry, Lanzhou 730000, China

^4^College of Pastoral Agriculture Science and Technology, Lanzhou University, Lanzhou 730000, China

^#^These authors contributed equally: Yingde Li, Yong Wei

*Corresponding authors: [duanty@lzu.edu.cn](mailto:duanty@lzu.edu.cn) (Tingyu Duan); [zhibiao@lzu.edu.cn](mailto:duanty@lzu.edu.cn) (Zhibiao Nan)

**Methods S1**

**Photosynthetic parameters**

Before harvesting, the photosynthetic parameters were measured, including net photosynthetic rate (Pn), intercellular carbon dioxide concentration (Ci), transpiration rate (E), and stomatal conductance (Gs). An open infrared portable gas-exchange fluorescence system (GFS-3000, Heinz Walz GmbH, Effeltrich, Germany) equipped with a 3 cm^2^ leaf chamber performed the measurements. Photosynthesis was measured from 9:00 a.m to 11:00 a.m. For each plant, four leaves of similar sizes and heights were selected for measurement from each plant and the middle leaflet measured four times. During the measurements, the temperature in the leaf chamber was 25℃, 50% relative humidity, 1000 ± 50 μmol m^−2^ s^−1^ photosynthetic photon flux density, and 410 ± 20 μmol mol^−1^ CO_2_ concentration. The leaf was placed in the chamber for 5 min until the readings for Pn and Gs were stable.

**Methods S2**

**Leaf samples processing for DNA extraction and amplicon sequencing**

First, rinse the dust from the leaves surface with sterile water. Then, leaves samples were subjected to sonication (15 min) and shaking (1 h, 200 rpm, 20℃) in 0.01 M sterile phosphate-buffered saline (PBS, 4℃), to dislodge the epiphytic microbes from the leaf surface. Then, the leaf samples were surface sterilized by consecutive immersion for 1 min in 75% ethanol, 3 min in 1% sodium hypochlorite, and 30 s in 75% ethanol, followed by three rinses with sterile water. Subsequently, the treated leaves were freeze-dried and homogenized.

**Methods S3**

**PCR reaction conditions**

Both the forward and reverse primers were tailed with sample-specific Illumina index sequences to allow for deep sequencing. The PCR was performed in a total reaction volume of 10 μl: DNA template 5-50 ng, *Vn F (10 μM) 0.3 μl, *Vn R (10 μM) 0.3 μl, KOD FX Neo Buffer 5 μl, dNTP (2 mM each) 2 μl, KOD FX Neo 0.2 μl, ddH_2_O up to 10 μl. Vn F and Vn R are selected according to the amplification area. After with initial denaturation at 95 °C for 5 min, followed by 25 cycles of denaturation at 95 °C for 30 s, annealing at 50 °C for 30 s, and extension at 72 °C for 40 s, and a final step at 72 °C for 7 min. The total of PCR amplicons were purified with Agencourt AMPure XP Beads (Beckman Coulter, Indianapolis, IN) and quantified using the Qubit dsDNA HS Assay Kit and Qubit 4.0 Fluorometer (Invitrogen, Thermo Fisher Scientific, Oregon, USA). After the individual quantification step, amplicons were pooled in equal amounts.

**Mycorrhizal colonization and plant growth**

**
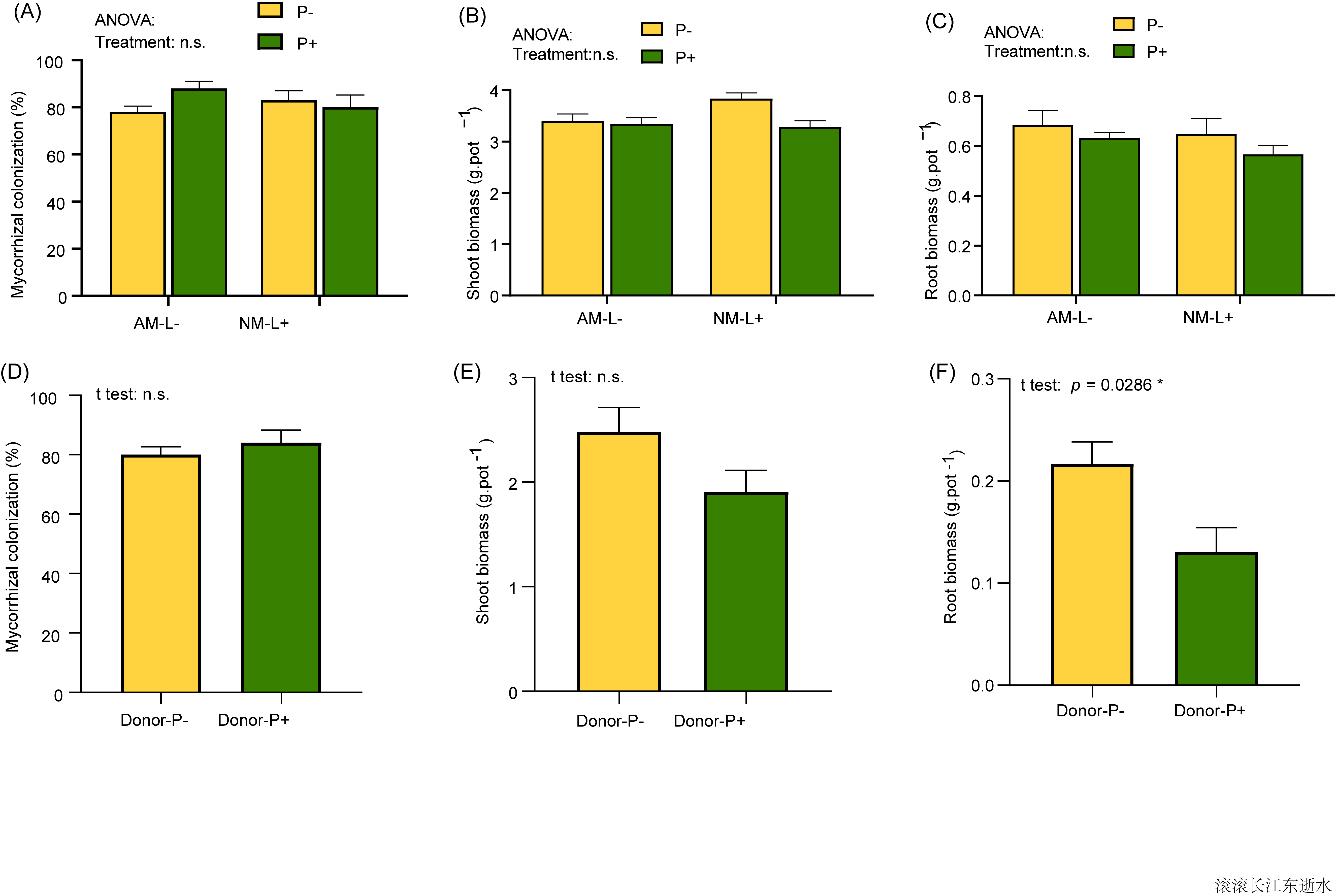
**

**Figure S1 Biomass and mycorrhizal colonization of white clover (*Trifolium repens*) and perennial ryegrass (*Lolium perenne*).** (A) Mycorrhizal colonization, (B) shoot biomass, and (C) root biomass of perennial ryegrass in linking donor white clover by common mycorrhizal networks (NM-L+) or un-linking (AM-L-). (D) mycorrhizal colonization, (E) shoot biomass, and (F) root biomass of donor white clover. P+ represents the donor clover infected with pathogen (*Stemphylium sarciniforme*), P- represents the donor clover un-infected with pathogen(*S. sarciniforme*). Values are presented as mean ± SEM of five replicates. Donor white clover data were analyzed using Student's t-test. * mean significant differences between the donor-P- and donor-P+ clover at *p* < 0.05. n.s. mean no significant differences.

**Plant photosynthesis**

**
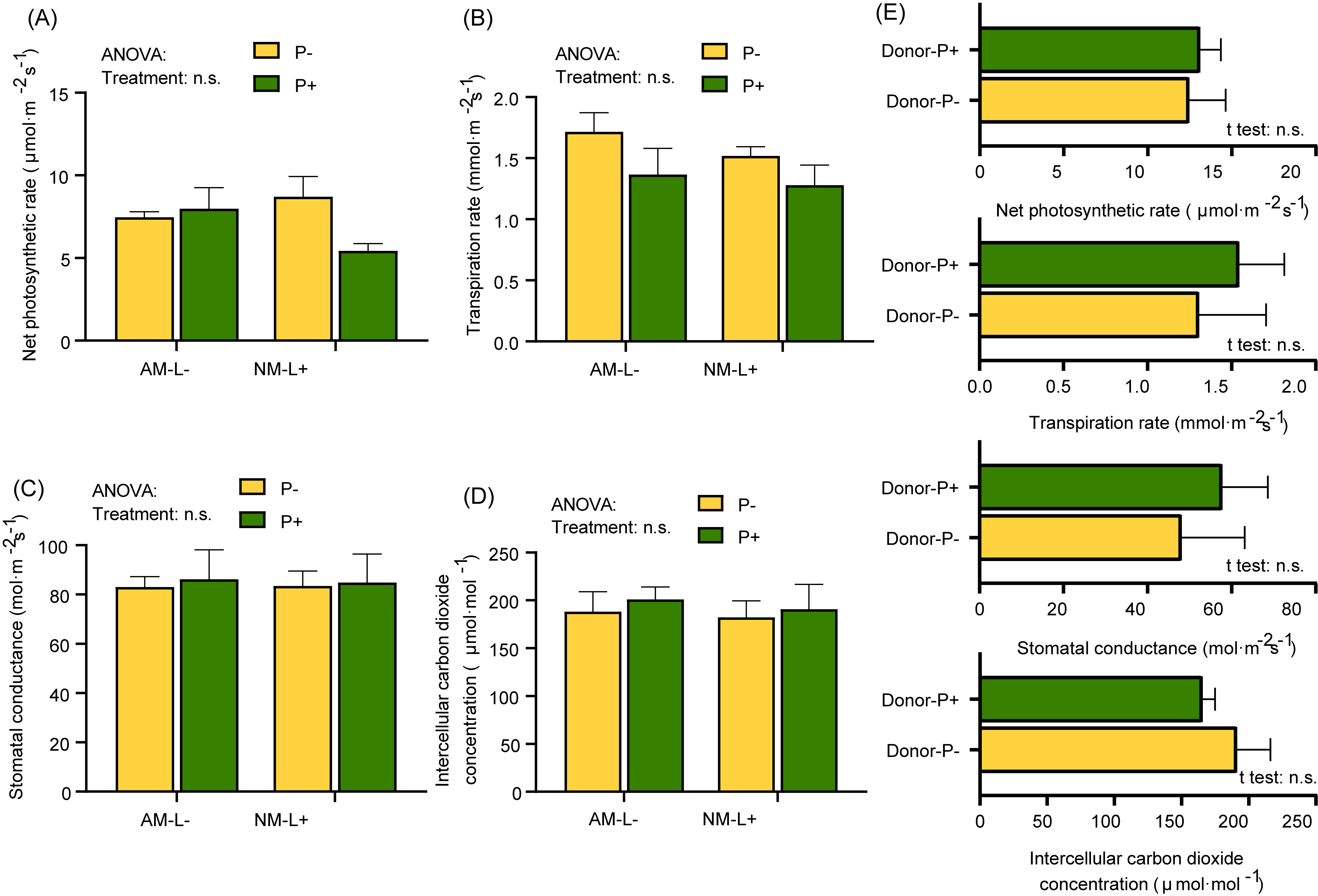
**

**Figure S2 Photosynthesis indices of white clover (*Trifolium repens*) and perennial ryegrass (*Lolium perenne*).** (A) Net photosynthetic rate (B) transpiration rate (C) stomatal conductance and (D) intercellular carbon dioxide concentration of perennial ryegrass in linking donor white clover by common mycorrhizal networks (NM-L+) or un-linking (AM-L-). (E) Net photosynthetic rate, transpiration rate, stomatal conductance, and intercellular carbon dioxide concentration of white clover. P+ represents the donor clover infected with pathogen(*Stemphylium sarciniforme*), P- represents the donor clover un-infected with pathogen (*S. sarciniforme*). Values are presented as mean ± SEM of five replicates. n.s. mean no significant differences.

**
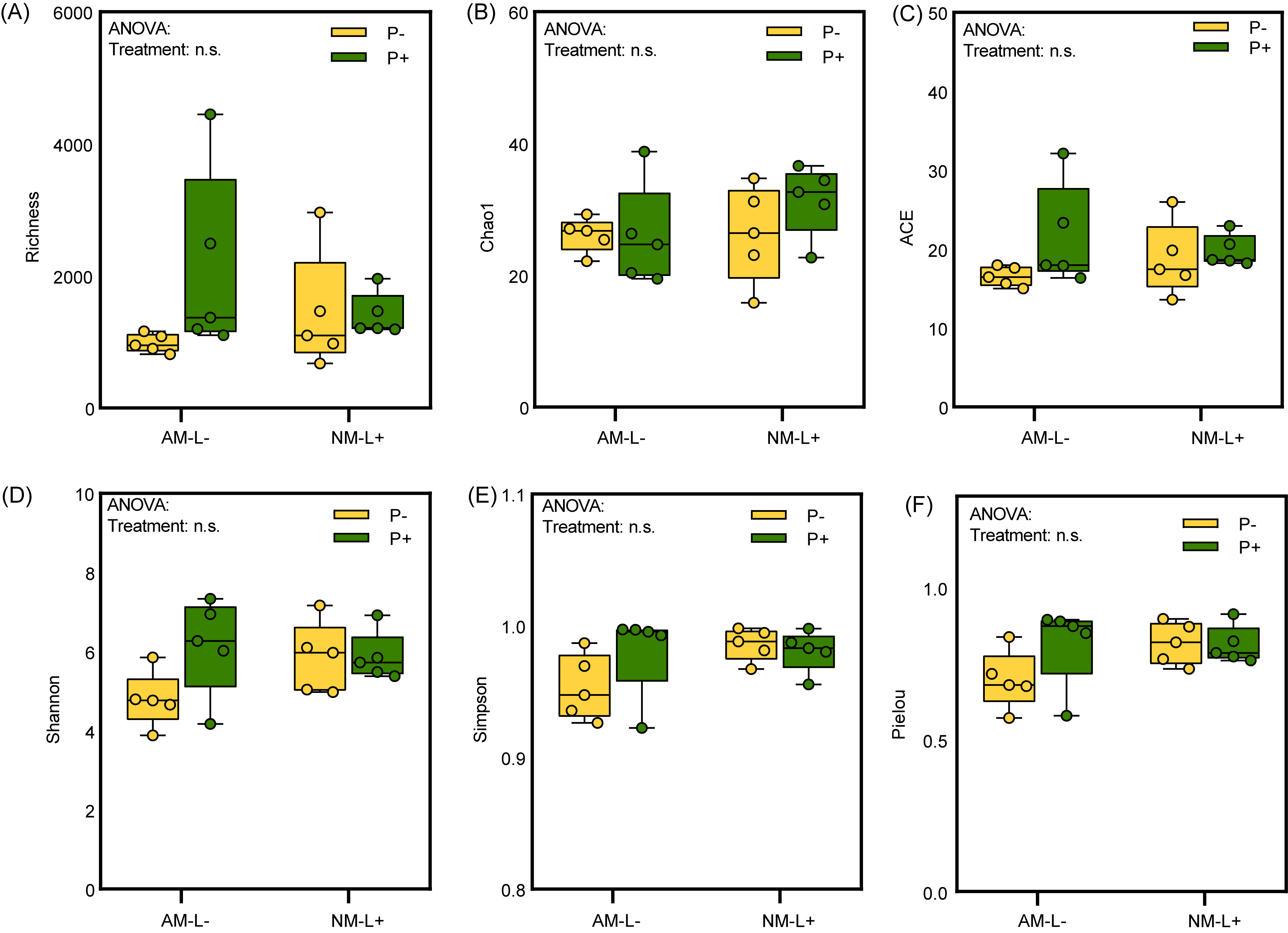
**

**Figure S3 The bacteria alpha diversity of perennial ryegrass (*Lolium perenne*).** (A) ASVs Richness (B) Chao1 index (C) ACE index (D) Shannon (E) Simpson and (F) Pielou of bacteria of perennial ryegrass in linking donor white clover (*Trifolium repens*) by common mycorrhizal networks (NM-L+) or un-linking (AM-L-). P+ represents the donor clover infected with pathogen (*Stemphylium sarciniforme*), P- represents the donor clover un-infected with pathogen (*S. sarciniforme*). n.s. mean no significant differences.


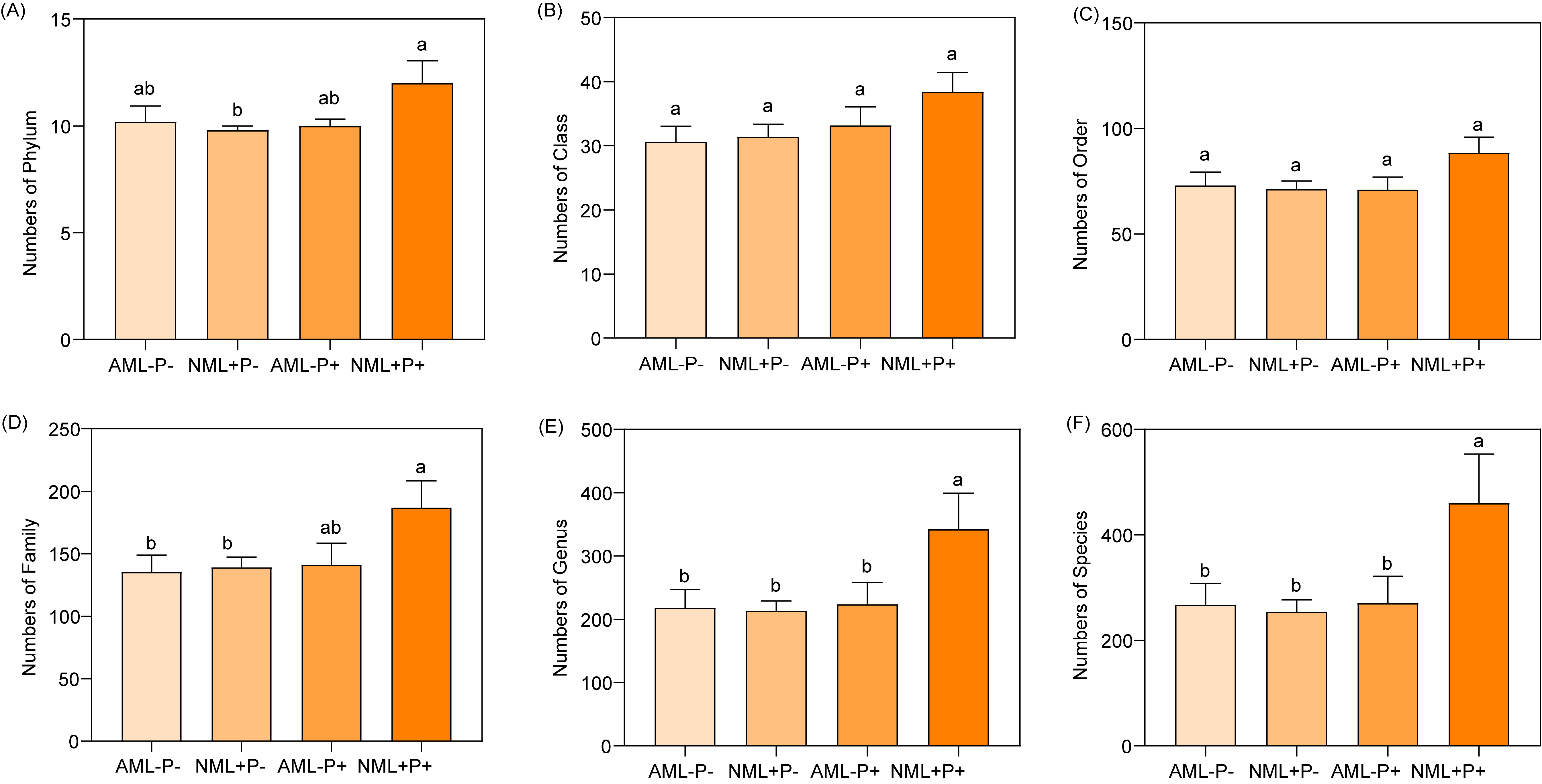


**Figure S4 Fungi diversity of perennial ryegrass (*Lolium perenne*).** (A-F) Diversity of fungi in ((A) Phylum (B) Class (C) Order (D) Family (E) Genus and (F) Species of perennial ryegrass in linking donor white clover (*Trifolium repens*) by common mycorrhizal networks (NM-L+) or un-linking (AM-L-). P+ represents the donor clover infected with pathogen (*Stemphylium sarciniforme*), P- represents the donor clover un-infected with pathogen (*S. sarciniforme*). Values are presented as mean ± SEM of five replicates. Different lowercase letters above the bars mean significant differences across groups and treatments at *p* < 0.05 by LSD test.

-
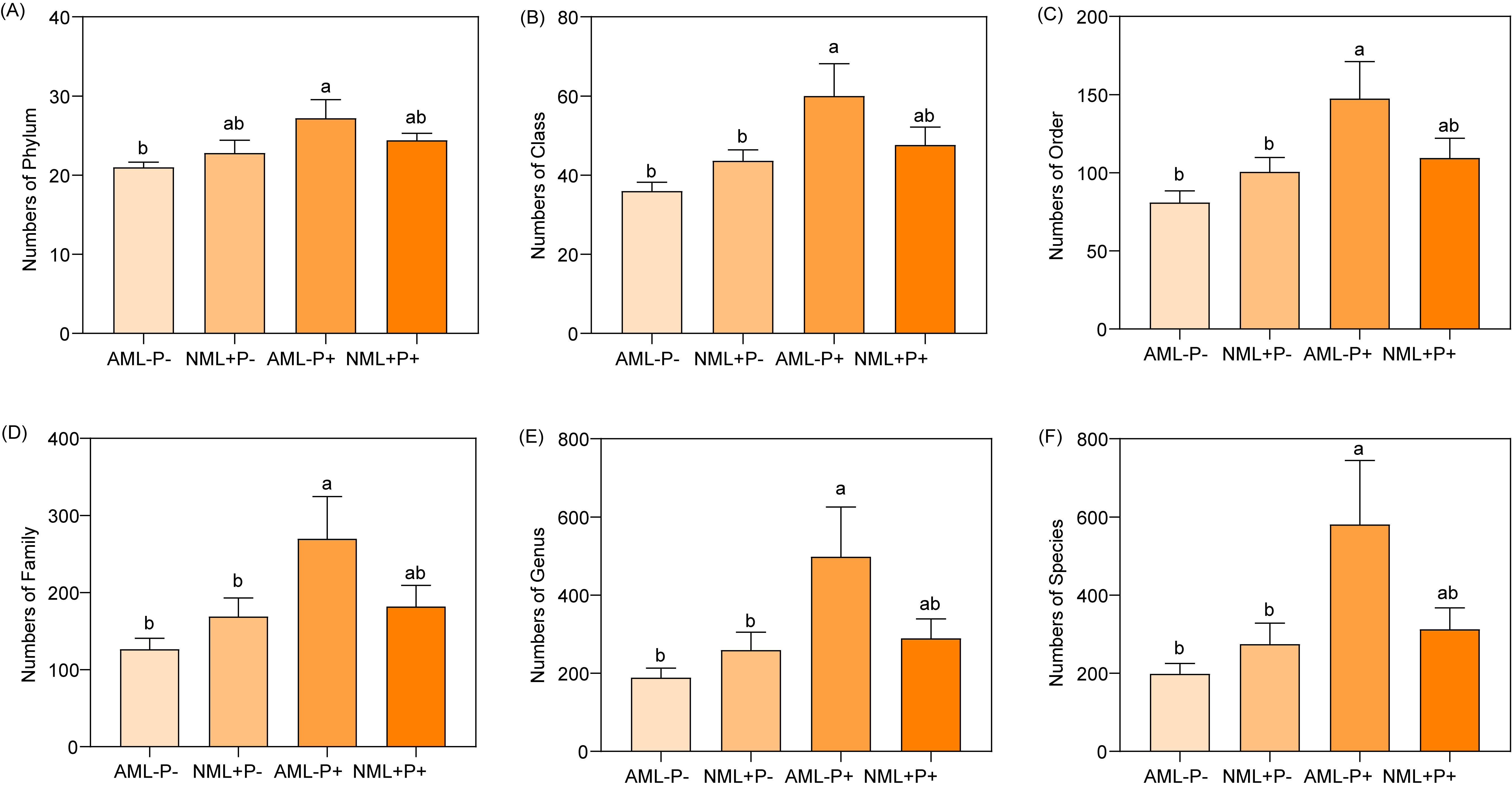


**Figure S5 Bacteria diversity of perennial ryegrass (*Lolium perenne*).** (A-F) Diversity of bacteria in (A) Phylum (B) Class (C) Order (D) Family (E) Genus and (F) Species of perennial ryegrass in linking donor white clover (*Trifolium repens*) by common mycorrhizal networks (NM-L+) or un-linking (AM-L-). P+ represents the donor clover infected with pathogen (*Stemphylium sarciniforme*), P- represents the donor clover un-infected with pathogen (*S. sarciniforme*). Values are presented as mean ± SEM of five replicates. Different lowercase letters above the bars mean significant differences across groups and treatments at *p* < 0.05 by LSD test.


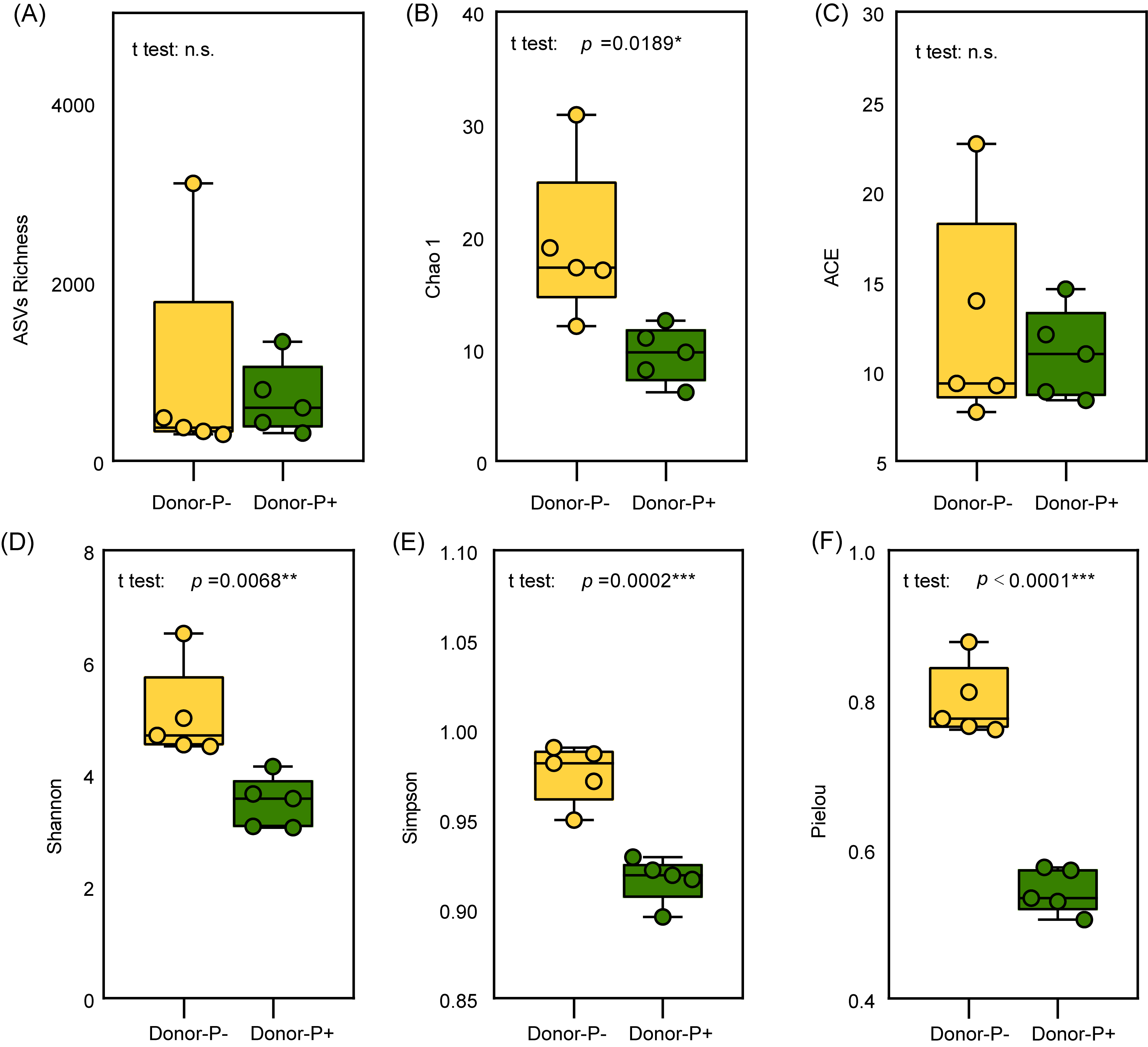


**Figure S6 The fungi alpha diversity of donor white clover (*Trifolium repens*).** (A) ASVs Richness (B) Chao1 index (C) ACE index (D) Shannon (E) Simpson and (F) Pielou of bacteria in white clover. P+ represents the donor clover infected with pathogen (*Stemphylium sarciniforme*), P- represents the donor clover un-infected with pathogen (*S. sarciniforme*). Donor white clover data were analyzed using Student's t-test. *, ** and *** mean significant differences between the donor-P- and donor-P+ clover at *p* < 0.05, *p* < 0.01, and *p* < 0.0001, respectively. n.s. mean no significant differences.


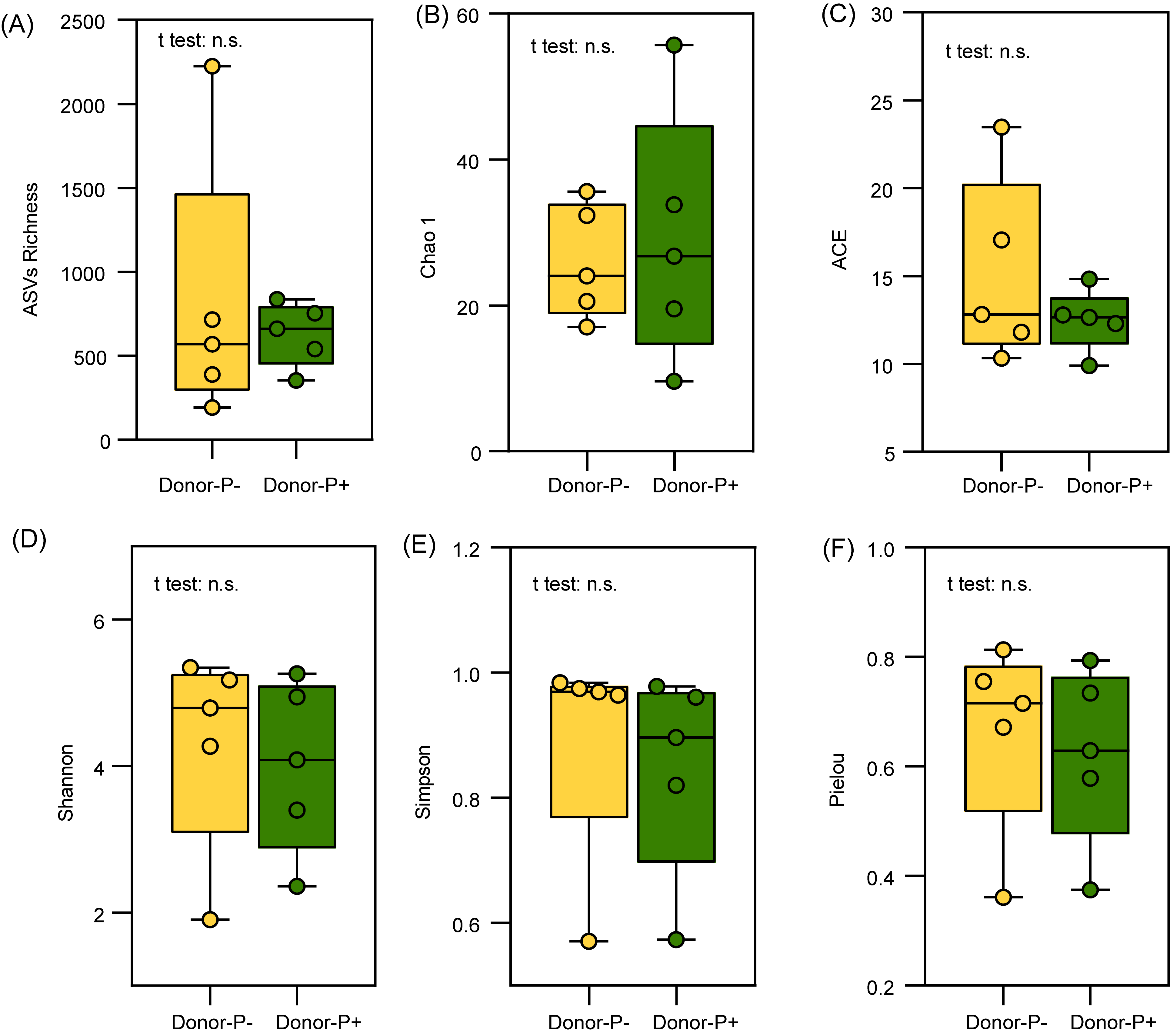


**Figure S7 The bacteria alpha diversity of donor white clover (*Trifolium repens*).** (A) ASVs Richness (B) Chao1 index (C) ACE index (D) Shannon (E) Simpson and (F) Pielou of fungal in white clover (*Trifolium repens*). P+ represents the donor clover infected with pathogen (*Stemphylium sarciniforme*), P- represents the donor clover un-infected with pathogen (*S. sarciniforme*). Donor white clover data were analyzed using Student's t-test. n.s. mean no significant differences.


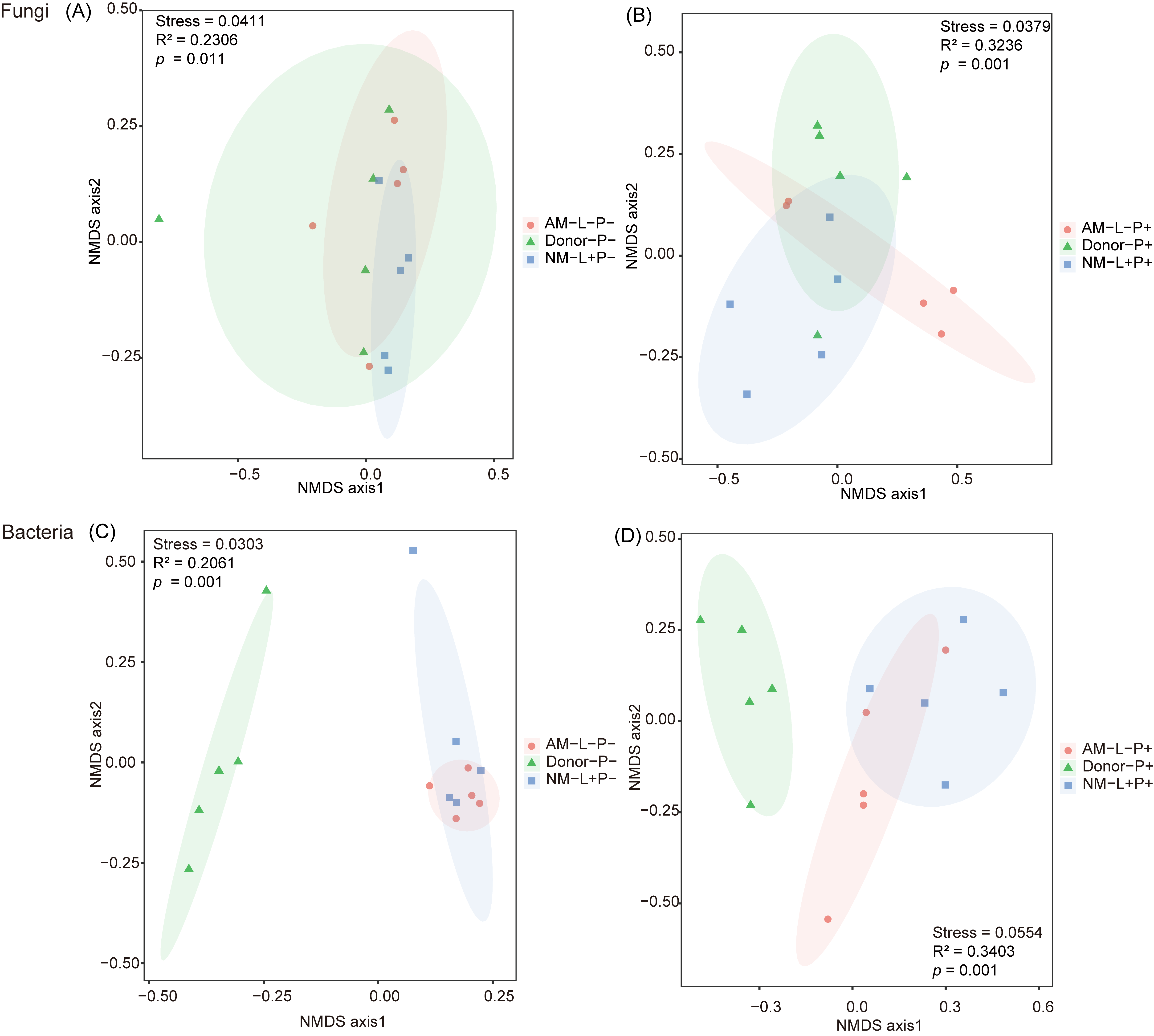


**Figure S8 The beta diversity of perennial ryegrass (*Lolium perenne*).** Non-metric multidimensional scaling (NMDS) of fungal (A, B) and bacterial (C, D) communities of perennial ryegrass linking donor white clover (*Trifolium repens*) by common mycorrhizal networks (NM-L+) or un-linking (AM-L-) and donor clover. P+ represents the donor clover infected with pathogen (*Stemphylium sarciniforme*), P- represents the donor clover un-infected with pathogen (*S. sarciniforme*).

**
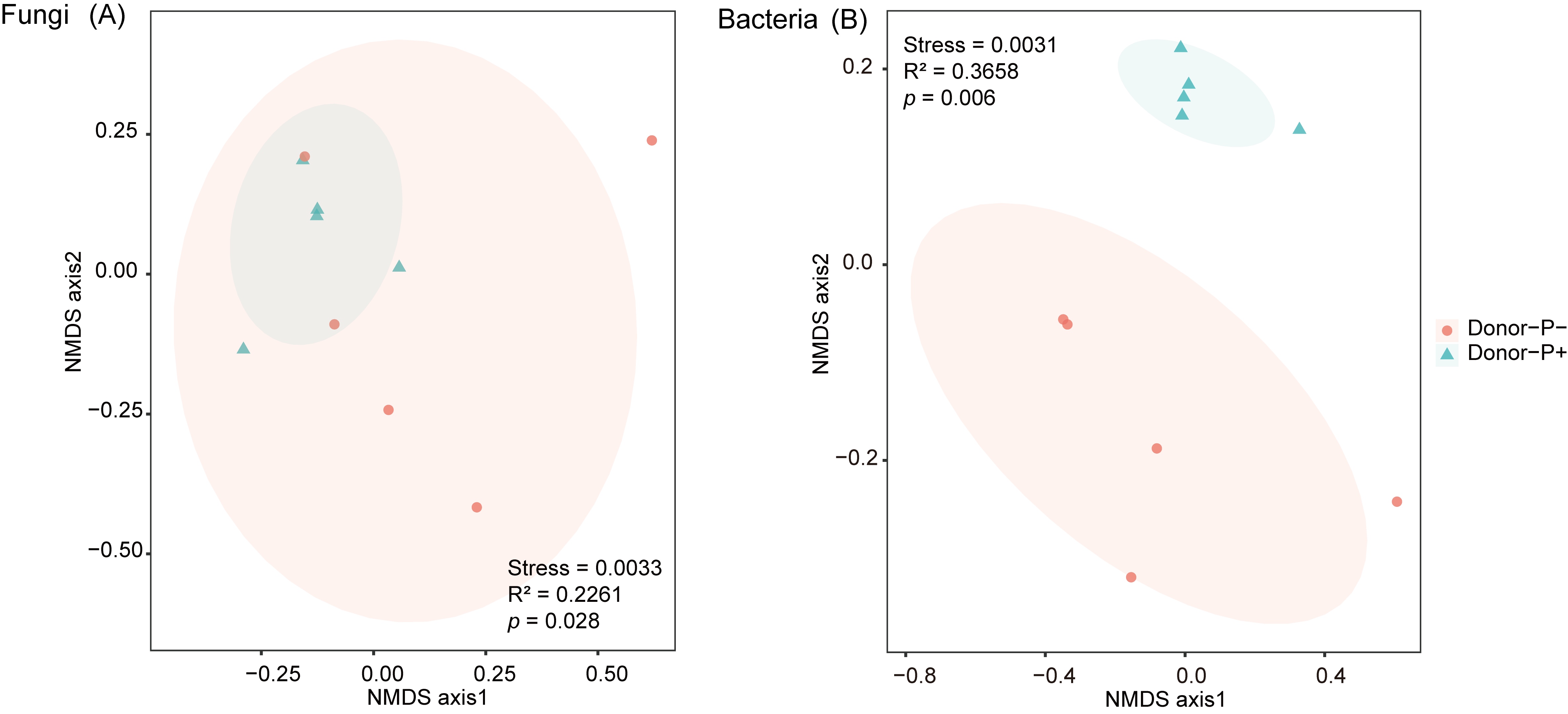
**

**Figure S9 The beta diversity of donor white clover (*Trifolium repens*).** Non-metric multidimensional scaling (NMDS) of fungal (A) and bacterial (B) communities in donor white clover. P+ represents the donor clover infected with pathogen (*Stemphylium sarciniforme*), P- represents the donor clover un-infected with pathogen (*S. sarciniforme*). The clustering is based on Bray-Curtis similarity and the resulting 2D stress for the best solution is 0.0033 (fungi) and 0.0031 (bacteria).


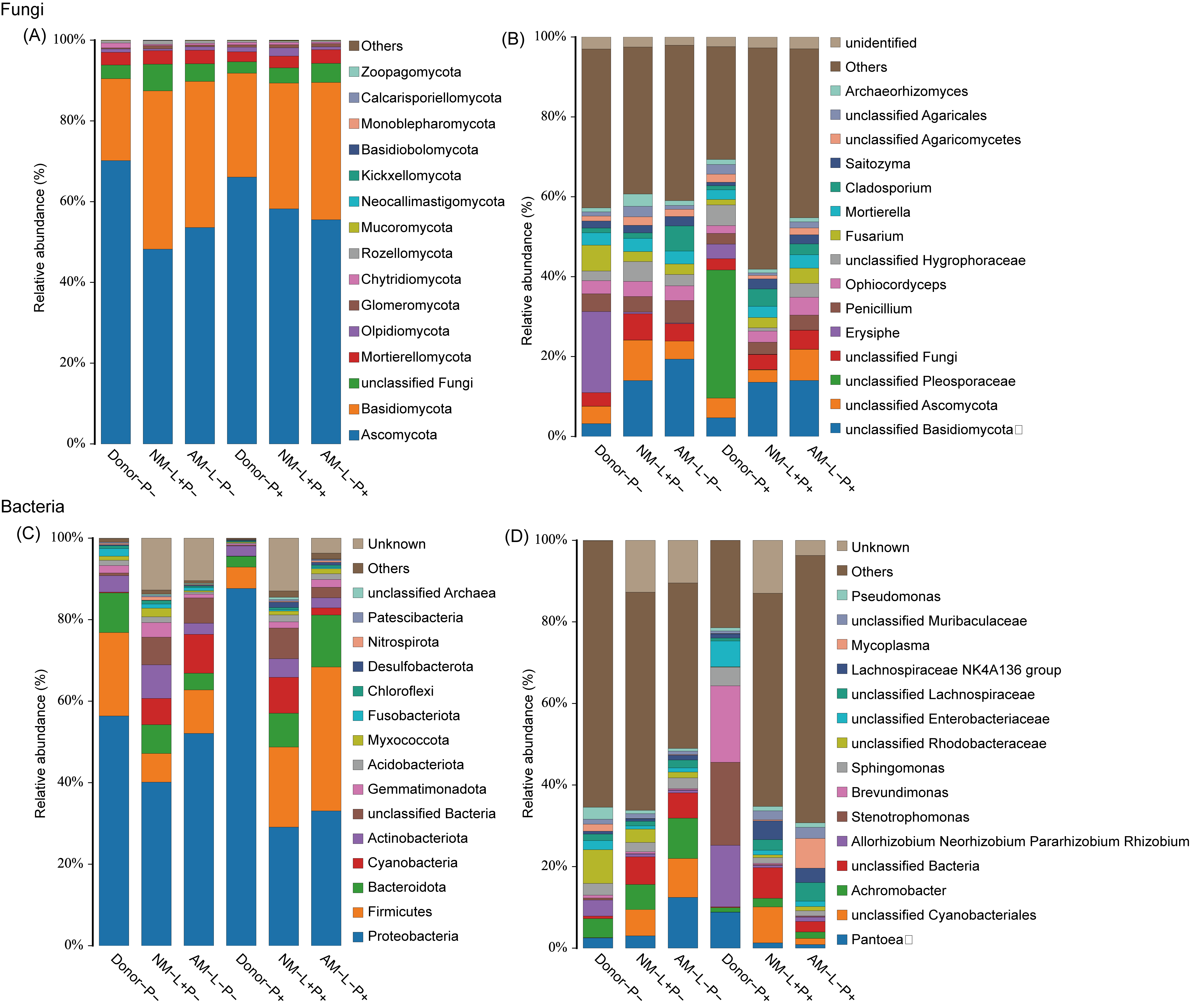


**Figure S10 Composition of the phyllosphere fungi and bacteria in perennial ryegrass (*Lolium perenne*) and white clover (*Trifolium repens*).** Fungi (A, B) and bacteria (C, D) relative abundance of ryegrass and clover at the phylum and genus level. All other less abundant or ambiguously assigned orders are grouped under ‘Others’


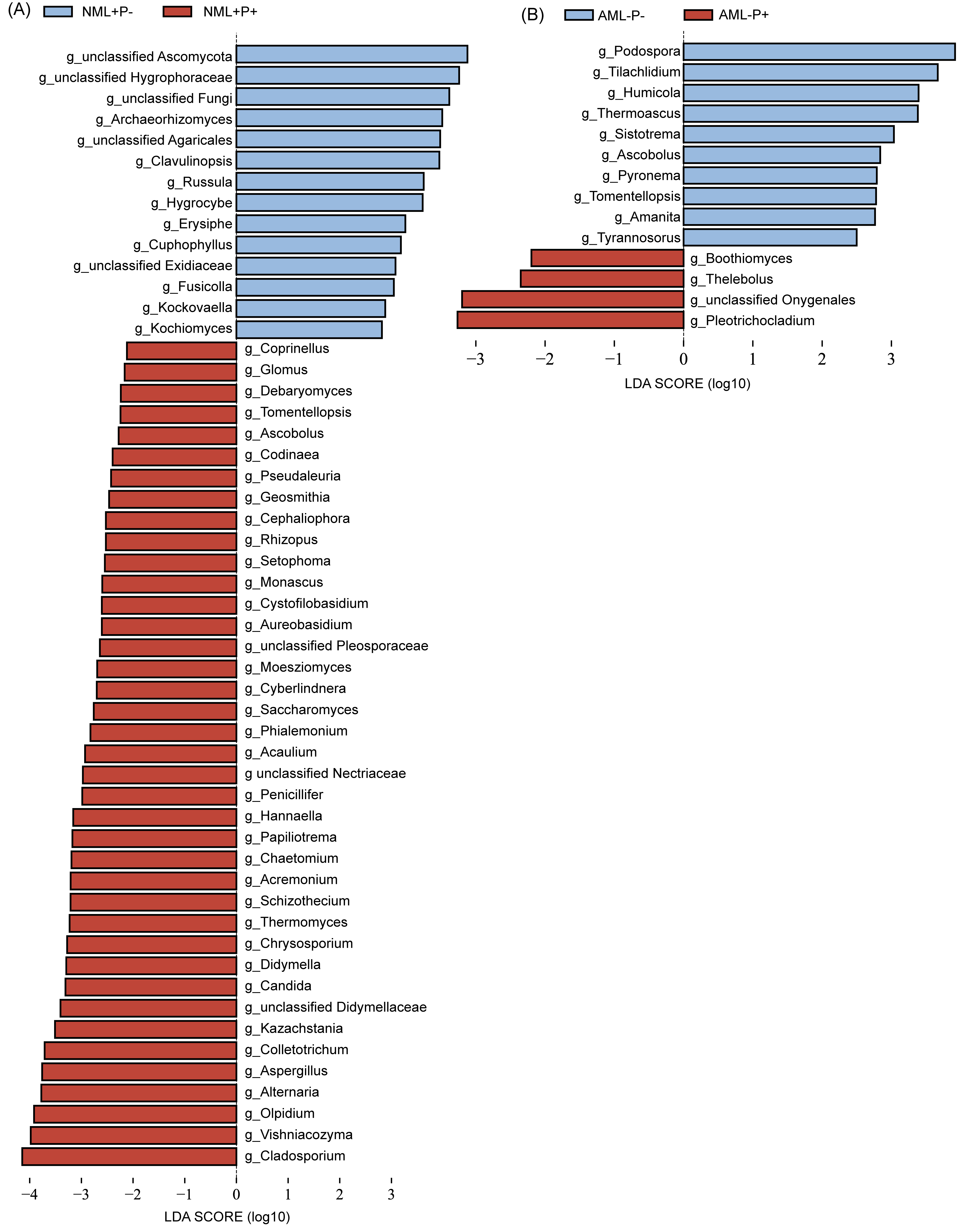


**Figure S11 LEfSe analysis of phyllosphere fungi of perennial ryegrass (*Lolium perenne*) linking donor white clover (*Trifolium repens*) by common mycorrhizal networks (NM-L+) or un-linking (AM-L-) in genus level.** P+ represents the donor clover infected with pathogen (*Stemphylium sarciniforme*), P- represents the donor clover un-infected with pathogen (*S. sarciniforme*). Values are presented as mean ± SEM of five replicates. The linear discriminant analysis (LDA) scores of the feature species driving the difference of fungal and bacterial community in different treatment.


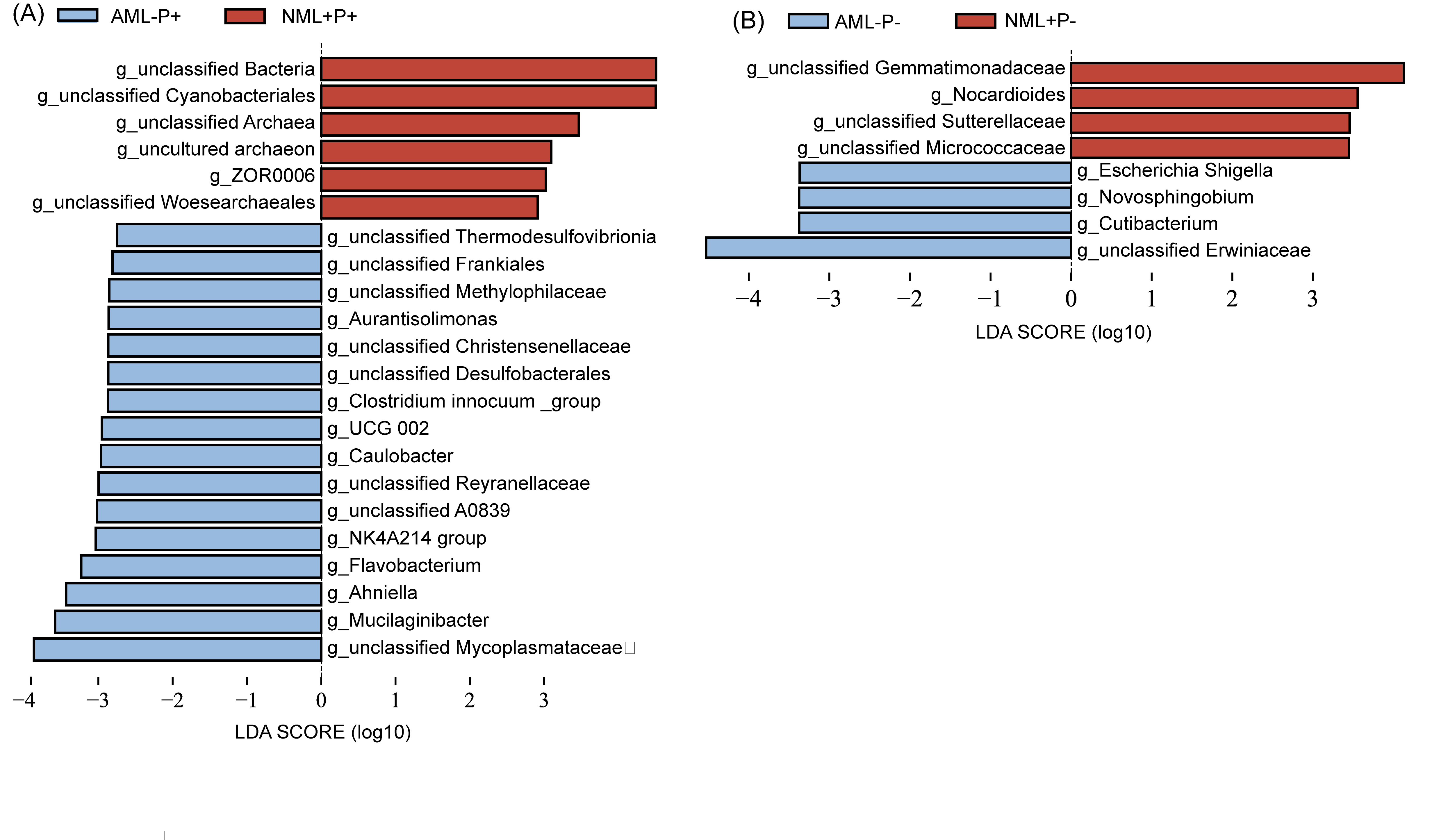


**Figure S12 LEfSe analysis of phyllosphere bacteria of perennial ryegrass (*Lolium perenne*) linking donor white clover (*Trifolium repens*) by common mycorrhizal networks (NM-L+) or un-linking (AM-L-) in genus level.** P+ represents the donor clover infected with pathogen (*Stemphylium sarciniforme*), P- represents the donor clover un-infected with pathogen (*S. sarciniforme*). Values are presented as mean ± SEM of five replicates. The linear discriminant analysis (LDA) scores of the feature species driving the difference of fungal and bacterial community in different treatment.


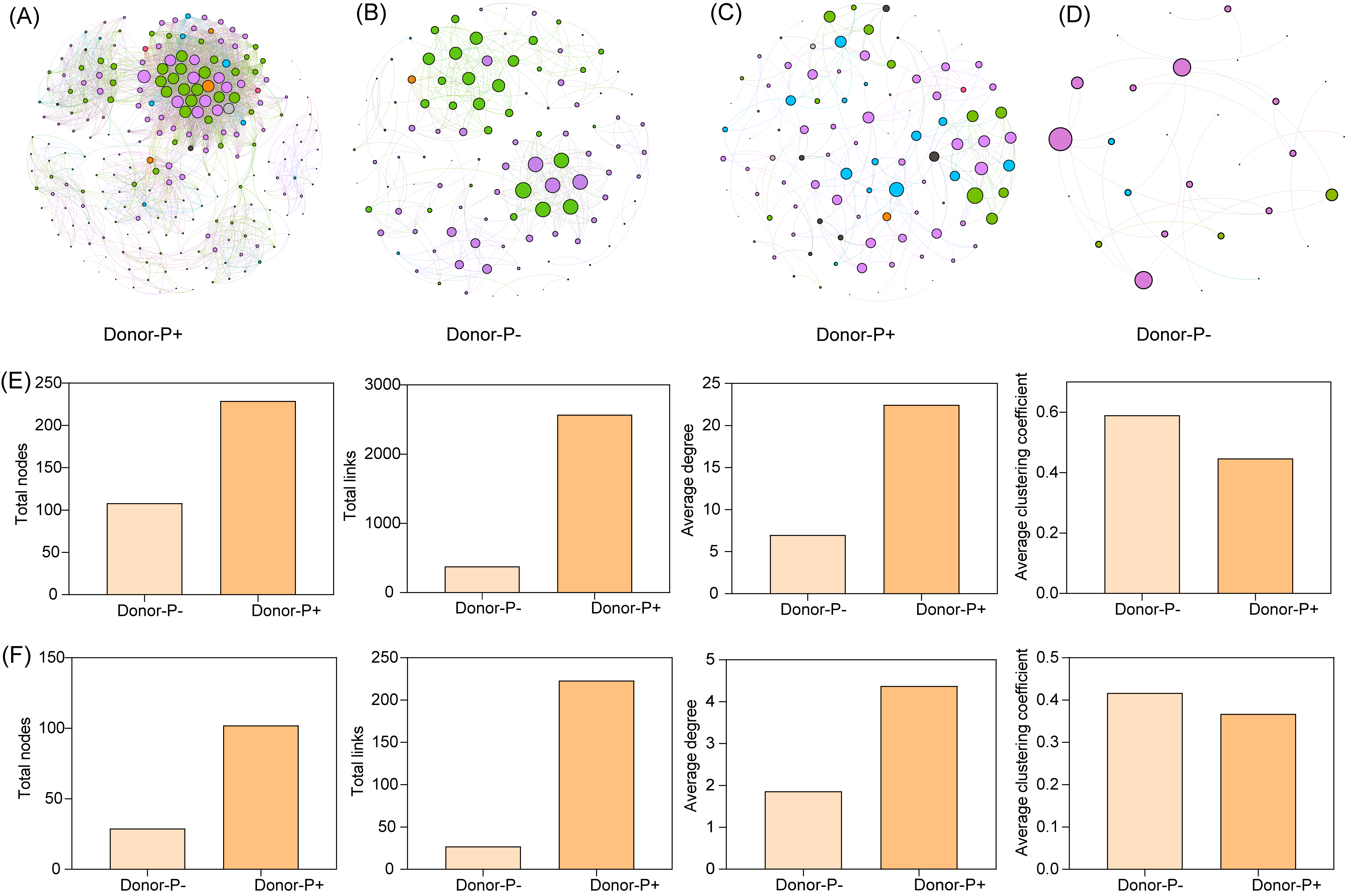


**Figure S13 Co-occurrence networks of donor white clover (*Trifolium repens*).** Co-occurrence networks of fungal (A, B) and bacterial (C, D) networks in donor white clover. (E) Topological properties of the fungal networks. (F) Topological properties of the bacterial networks. P+ represents the donor clover infected with pathogen (*Stemphylium sarciniforme*), P- represents the donor clover un-infected with pathogen (*S. sarciniforme*).


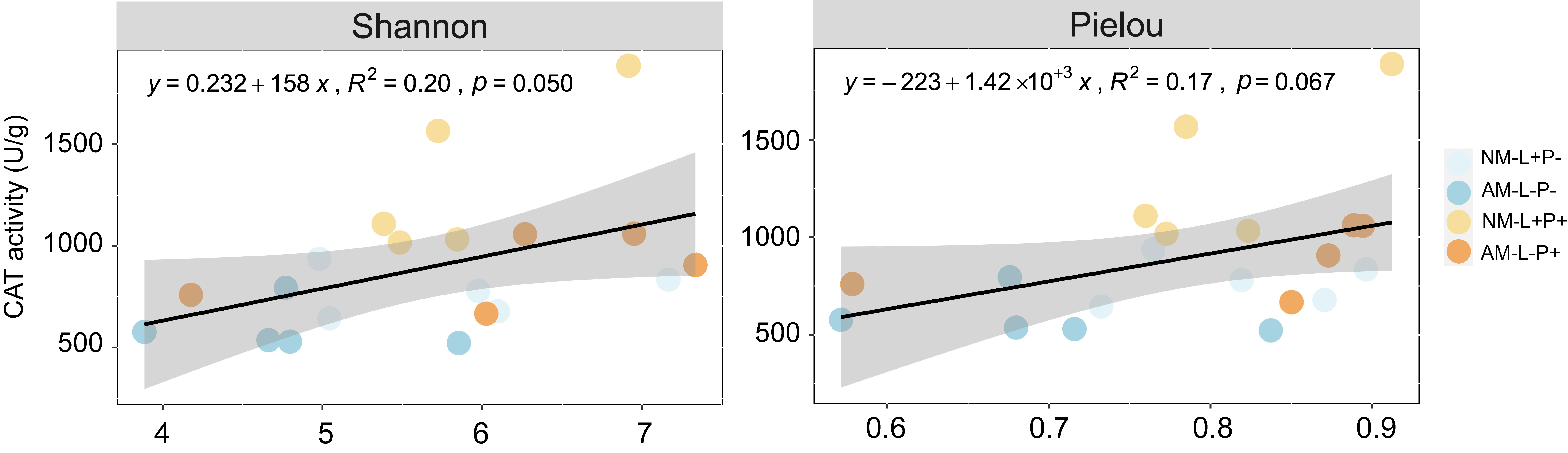


**Figure S14 Regression equations between catalase activity and bacteria alpha diversity of receiver perennial ryegrass (*Lolium perenne*).** The coefficient of determination (R^2^) and statistical significance are shown.

**
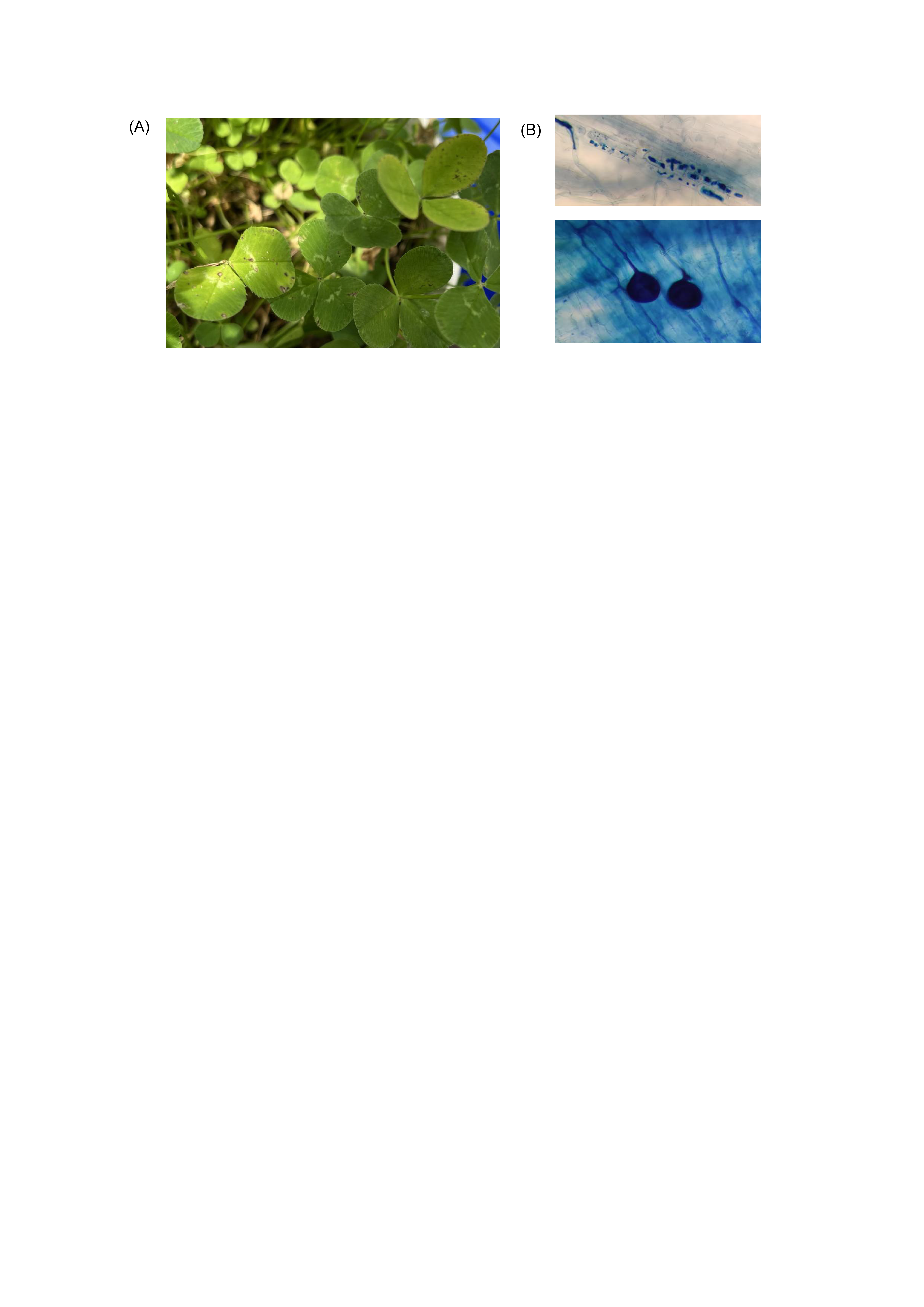
Figure S15 The pictures of diseased leaves of donor white clover (*Trifolium repens*) mycorrhizal colonization.** (A) diseased leaves of donor white clover and (B) mycorrhizal colonization.

**
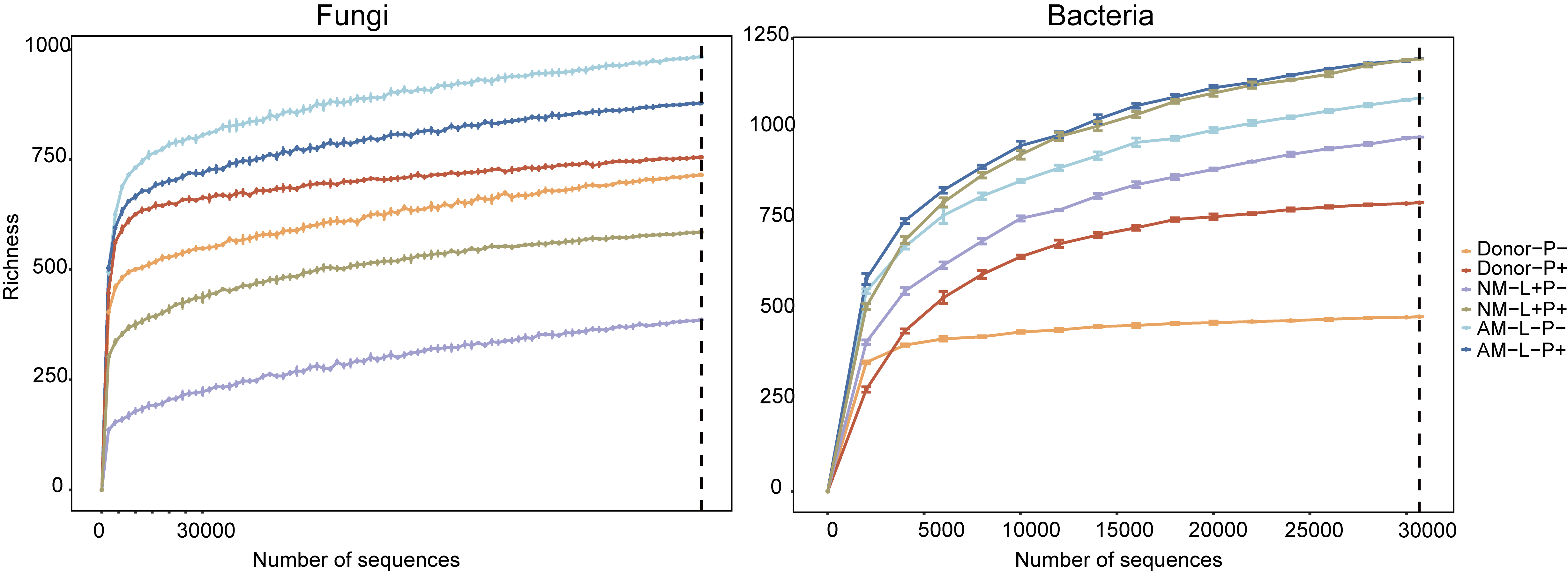
**

**Figure S16 Dilution curves of the phyllosphere fungi and bacteria in white clover (*Trifolium repens*) and perennial ryegrass (*Lolium perenne*).**
